# *Cryptococcus neoformans* serotype A virulence and pathogenicity are capsular glucuronoxylomannan (GXM) motif composition dependent

**DOI:** 10.1128/mbio.02646-25

**Published:** 2025-09-29

**Authors:** Samuel Rodrigues Dos Santos Junior, Piotr R. Stempinski, Marcelo Valdemir de Araujo, Kevin Rojas, Gracen R. Gerbig, Livia C. Liporagi Lopes, Daniel F. Q. Smith, Maggie P. Wear, Arturo Casadevall

**Affiliations:** 1 W. Harry Feinstone Department of Molecular Microbiology and Immunology, The Johns Hopkins Bloomberg School of Public Health, Baltimore, Maryland, USA; 2 Departamento de Imunologia, Universidade de São Paulo, Instituto de Ciências Biomédicas, São Paulo, Brazil; 3 Departamento de Análises Clínicas e Toxicológicas, Faculdade de Farmácia, Universidade Federal do Rio de Janeiro, Rio de Janeiro, Brazil; University of Florida College of Dentistry, Gainesville, Florida, USA

**Keywords:** *Cryptococcus neoformans*, capsular polysaccharide, GXM, virulence, glucuronoxylomannan, motifs, pathogenesis, capsule, extracellular polysaccharide, exopolysaccharide

## Abstract

The *Cryptococcus neoformans* capsular extracellular polysaccharide (EPS) plays an essential role in virulence and host immunomodulation. In this study, we analyzed the virulence of four serotype A *C. neoformans* strains with different polysaccharide structures: two multi-motif (H99 and KN99α) and two single-motif (Mu-1 and ATCC-24064 (24064)). Mice were infected by the intranasal (IN) or intravenous (IV) route. Survival, fungal load (colony-forming unit), cell lung population, cytokines, and histology were analyzed. No differences in cell size or capsule size were found between the four strains. However, polysaccharide sizes were highly variable, and no significant differences were observed for all other virulence factors. Nitrite production in cell culture dendritic cells was reduced for the H99 or KN99α strains of EPS. The reactive oxygen species in cell culture M1 macrophages and dendritic cells were increased for the H99 or KN99α strain’s EPS. Cytokines differed from the IN and IV infections. Lung histology showed increased inflammatory cell presence after infection with Mu-1 or 24064 strains, with no cryptococcal cells observed in the lung parenchyma or alveoli, whereas the opposite pattern was seen after challenges with H99 or KN99α. There were differences in lung neutrophil infiltration between the analyzed strains, and a higher presence of activated macrophages and CD4+ lymphocytes for the Mu-1 or 24064 strains. Polysaccharides from single and multi-motif strains elicited different effects on macrophage-like cells *in vitro*. These results suggest that variation in polysaccharide composition and structure can translate into differences in virulence and pathogenicity.

**IMPORTANCE:**

Cryptococcosis is a systemic fungal infection that causes approximately 1 million cases globally, leading to approximately 625,000 annual deaths. Two species are responsible for the majority of cases, *Cryptococcus neoformans* and *Cryptococcus gattii*. *C. neoformans* usually causes disease in immunosuppressed hosts, whereas *C. gattii* can infect and cause disease in immunocompetent hosts. The capsule of *Cryptococcus* spp. is one of its major virulence factors, due to its immunomodulation abilities. The capsule is composed of three major different molecules: glucuronoxylomannan (GXM), galactoxylomannan (GalXM), and mannoproteins. GXM is composed of six different structures called motifs. The exact mechanism and structures associated with its immunomodulation are still not well elucidated. Here, we looked at different immune responses based on the capsule composition. Our results strongly suggest that the capsule motif composition can affect the virulence, pathogenicity, and immunomodulatory capabilities of the *Cryptococcus* capsule.

## INTRODUCTION

Cryptococcosis is a systemic fungal infection caused by yeasts of the genus *Cryptococcus* (1). Most cases of cryptococcosis are caused by two species, *Cryptococcus neoformans* and *Cryptococcus gattii* (2–4). The infection starts when the host inhales fungal propagules (desiccated yeast or spores), and after inhalation, the propagules are carried to the lungs, but some propagules may stay adhered in the upper airways (5–8). After the contact with the host, the propagules become active yeast (2–4).

*C. neoformans* has a polysaccharide capsule composed of glucuronoxylomannan (GXM), galactoxylomannan (GalXM), and mannoproteins (9–11). The polysaccharide capsule is one of the most important cryptococcal virulence factors alongside melanin (12, 13). GXM is structurally diverse with polysaccharide molecules composed of six different conformational structures (motifs) based on the amount and ligand configuration of its subunits (α−1,3-mannose backbone, β-1,2- and β-1,4-xylose and β-1,2-glucuronic acid). Different motifs and proportions of each motif are found composing the capsule (2–4). One of the first attempts to identify the etiological agent causing cryptococcal infections was based on GXM motif-specific antibodies present in the sera collected from infected hosts (14, 15). Five serotypes were identified from serology: serotype A for *C. neoformans* var. *grubii*, serotype D for *C. neoformans* var. *neoformans*, serotype AD for hybrids of serotypes A and D, and serotypes B and C for *C. neoformans* var. *gattii* (14, 15). Currently, more than 30 species in the *Cryptococcus* genus are recognized, and their classification is based on molecular types (16, 17). The *Cryptococcus* capsule is directly connected with its ability to survive and consequently spread through the host body (8, 18). The first mechanism is based on the capsule’s ability to shield pathogen-associated molecular patterns (PAMPs) and microbe-associated molecular patterns (MAMPs) to avoid recognition by immune cells and phagocytosis (19). The second mechanism is related to the immunological modulation caused by GXM (19–22). GXM can modulate the immune response from a Th1 and Th17 protective immunity to a Th2 non-protective immunity. This modulation occurs by the stimulation of some anti-inflammatory cytokines, like IL-4 and IL-6, and polarization of M2 macrophages (22, 23). The capsule also allows yeast survival inside macrophages and dendritic cells (DCs) by buffering lysosomal pH (24–26). Another pathogenic mechanism of the cryptococcal capsule is the release of capsular content to the surrounding environment and tissues (22, 27, 28). The capsular content released is called extracellular polysaccharide or exopolysaccharide (EPS), whereas the polysaccharide attached to the cell is called capsular polysaccharide (CPS) (22, 27, 28). This shedding mechanism may be responsible for the increase in the intracranial pressure (ICP) in hosts with cryptococcal meningoencephalitis (22, 27, 28).

Although the capsule is acknowledged to be the major virulence factor, there are major differences in the polysaccharide structure, but their contributions to virulence have not been studied. Consequently, we analyzed the differences in virulence and pathogenicity of four *C. neoformans* serotype A strains, comparing the multi-motif strains H99 and KN99α with the single-motif strains Mu-1 and 24064. We observed differences in fungal load and survival rates using three different models of infection—intranasal (IN), intravenous (IV), and intratracheal (IT)—and also observed differences in the cytokine levels, lung cell population of infected mice, and histological findings, although other virulence factors were similar between the different strains.

## RESULTS

### Phenotypic characterization of serotype A strains

**Fig 1:**
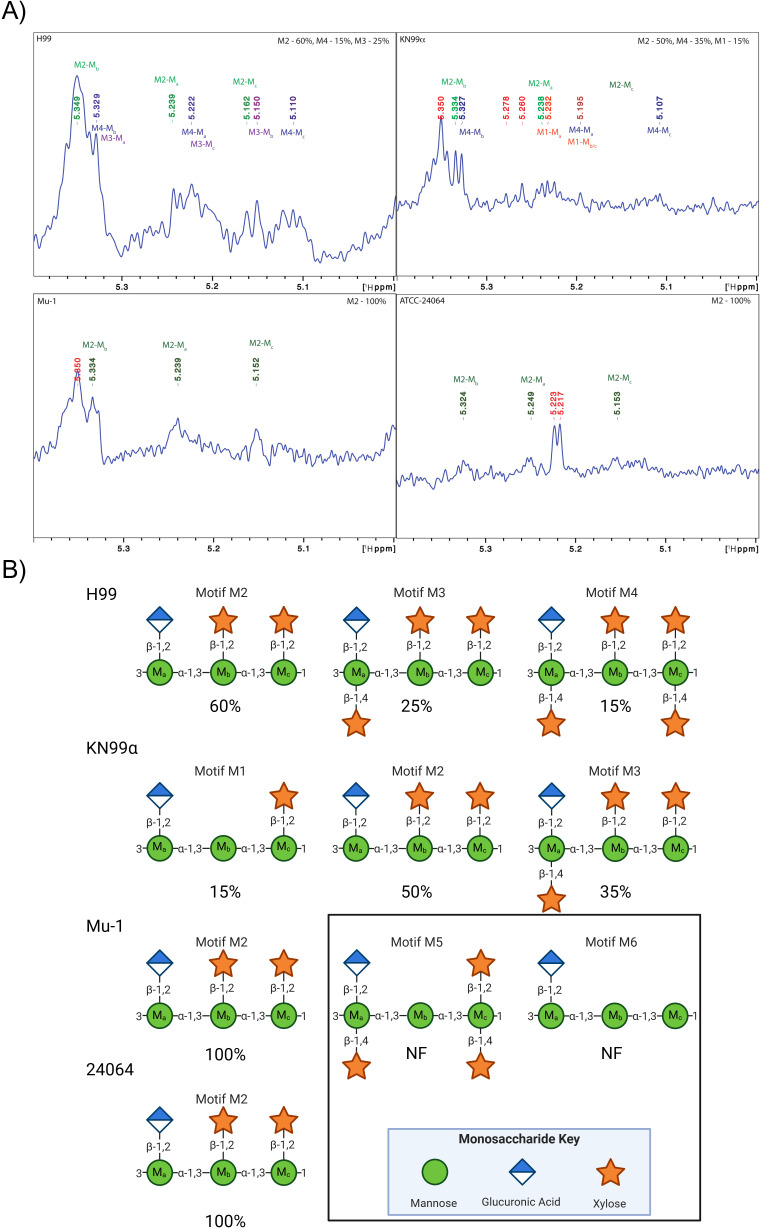
GXM motif prevalence in EPS from the different serotype A strains of *C. neoformans* determined by proton NMR. EPS was produced after the growth of different serotype A strains of *C. neoformans* in MM for 1 week at 30°C under agitation (150 rpm). (**A**) Representative 1D [1H] NMR spectra of the SRG region with peak chemical shift assignments for each GXM motif. (**B**) Strain GXM motif makeup as determined by the integral percentage of motif assigned chemical shifts. Sequence and linkage of GXM motif repeats using glycobiology illustrative shorthand. NF = not found.

**Fig 2:**
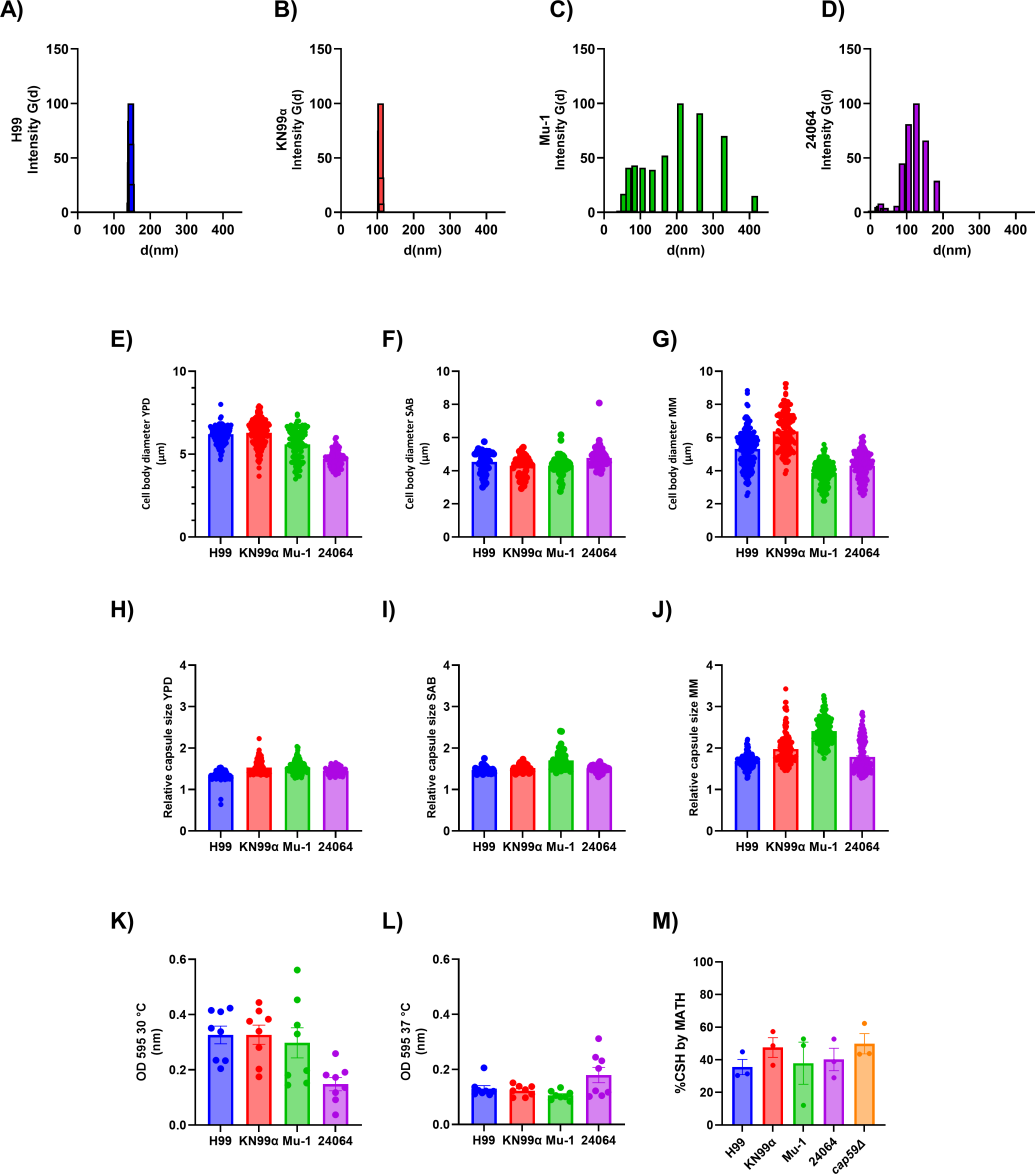
Phenotypic characterization of the EPS from serotype A strains of *C. neoformans*. After growth of different serotype A strains of *C. neoformans* in MM for 1 week at 30°C under agitation (150 rpm), the media were filtered with a 0.22 µm size filter. (**A**) Analysis of H99 EPS size and polydispersity by DLS (*n* = 5 per group). (**B**) Analysis of KN99α EPS size and polydispersity by DLS (*n* = 5 per group). (**C**) Analysis of Mu-1 EPS size and polydispersity by DLS (*n* = 5 per group). (**D**) Analysis of 24064 EPS size and polydispersity by DLS (*n* = 5 per group). (**E**) YPD Cell body size (*n* = 50 per group). (**F**) SAB cell body size (*n* = 50 per group). (**G**) MM cell body size (*n* = 50 per group). (**H**) YPD Capsule relative size (*n* = 50 per group). (**I**) SAB capsule relative size (*n* = 50 per group). (**J**) MM capsule relative size (*n* = 50 per group). (**K**) Biofilm formation at 30°C (*n* = 8 per group). (**L**) Biofilm formation at 37°C, respectively (*n* = 8 per group). (**M**) Percentage of cell surface hydrophobicity (*n* = 3 per group). No significant statistics were observed (*t*-test and/or ANOVA). Multiple comparisons were corrected by Šídák’s multiple comparisons test.

GXM motif repeat expression was determined by 1D proton nuclear magnetic resonance (NMR) of EPS (Fig. 1A; Table S2). Here, we describe for the first time that *C. neoformans* strain H99 expresses 60% M2, 25% M3, and 15% M4 (Fig. 1B), whereas *C. neoformans* strain KN99α expresses 15% M1, 50% M2, and 35% M4 (Fig. 1B). We also confirmed the single M2 motif composition of *C. neoformans* strain Mu-1 and 24064 (Fig. 1A), as previously reported by Cherniak (29). Analysis of EPS by dynamic light scattering (DLS) (Fig. 2A through D) revealed that the multi-motif strains H99 and KN99α had a smaller EPS size and a lower size population distribution based on the polydispersity index (PDI) analysis, 0.114 and 0.136, respectively, whereas the single-motif strains Mu-1 and 24064 had a larger EPS size and higher PDI 0.393 and 0.312, respectively.

No difference was observed in the cell size (Fig. 2E through G; Fig. S1) between the four strains using different growth media (YPD, SAB, or MM). *C. neoformans* cells cultivated in MM had a slightly larger capsule (Fig. 2H, I, and J; Fig. S1). No difference was observed in biofilm formation between the four strains at 30°C and 37°C (Fig. 2K and L). Comparison of the cell surface hydrophobicity (Fig. 2M) of the four serotype A strains and one acapsular mutant (H99, KN99α, Mu-1, 24064, and *cap59*∆) revealed no significant differences. All capsular *C. neoformans* serotype A strains (H99, KN99α, Mu-1, and 24064) had the same level of GXM shedding (EPS) after 1 week of culture in MM (Fig. S2), and no significant amount of protein was observed in the EPS (Fig. S3).

### Expression of virulence factors by the four serotype A strains

**Fig 3:**
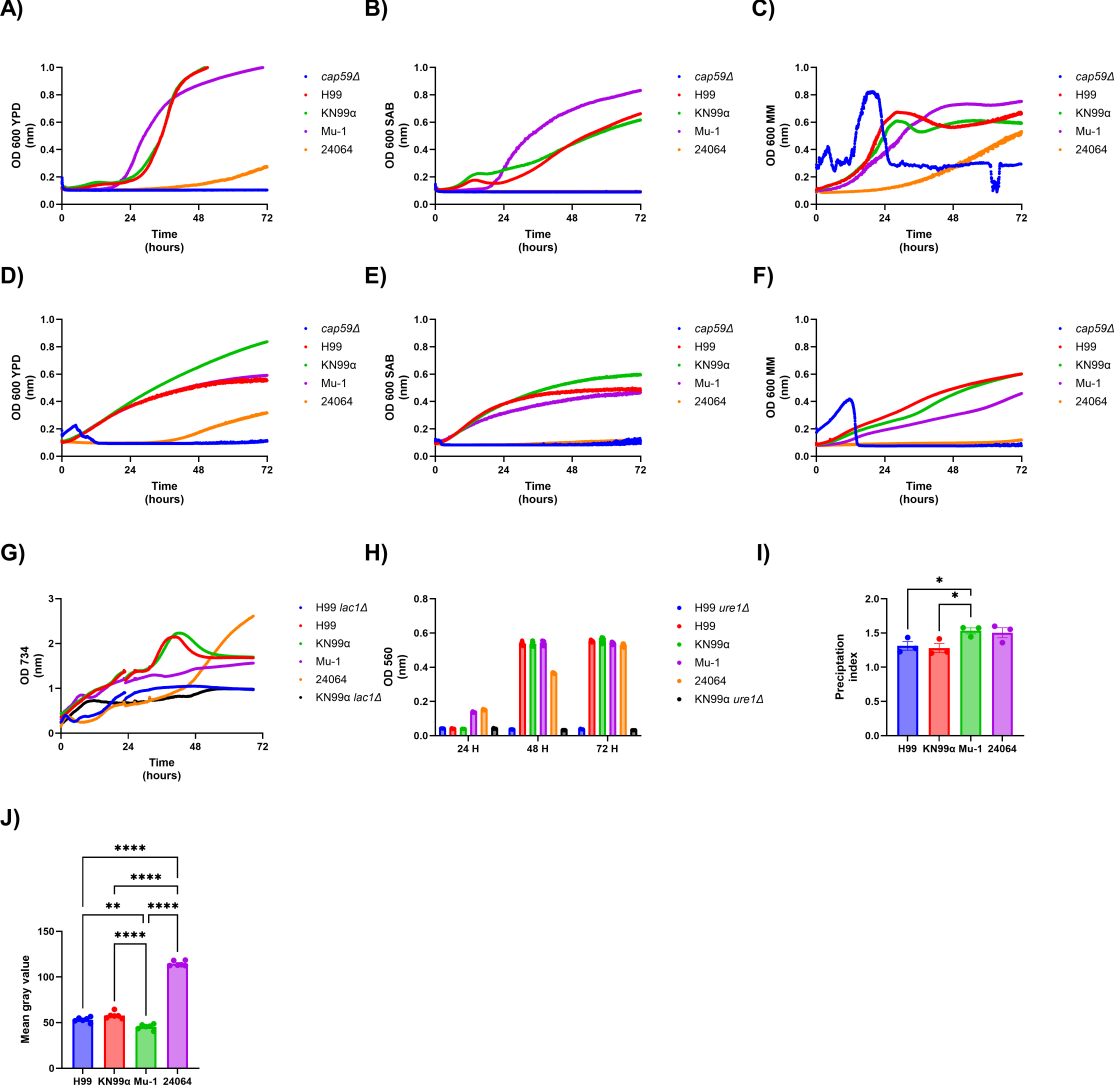
Different virulence factors of *C. neoformans*. Serotype A strains of *C. neoformans* (*cap59*∆, H99, KN99α, Mu-1, or 24064) were grown in different culture media (YPD, SAB, MM, or UB) and at different temperatures (30°C or 37°C), and their main virulence mechanisms were evaluated. (**A**) YPD 30°C growth rate (*n* = 4 per group). (**B**) SAB 30°C Growth rate (*n* = 4 per group). (**C**) MM 30°C growth rate (*n* = 4 per group). (**D**) YPD 37°C growth rate (*n* = 4 per group). (**E**) SAB 37°C growth rate (*n* = 4 per group). (**F**) MM 37°C growth rate (*n* = 4 per group). The unusual shape of growth curves for *cap59*Δ reflects the fact that under certain conditions, it aggregates. (**G**) Laccase activity (*n* = 6 per group). H99 and KN99α laccase knockout (*lac1*Δ) were used as a negative control. (**H**) Urease activity (*n* = 9 per group), H99 and KN99α urease knockout (*ure1*Δ) were used as negative control. (**I**) Phospholipase activity (*n* = 3 per group). (**J**) Mean gray value of the melanin production (*n* = 6 per group). * = *P* < 0.05, ** = *P* < 0.01, **** = *P* < 0.0001 (*t*-test and or ANOVA). Multiple comparisons were corrected by Šídák’s multiple comparisons test.

The growth rate was evaluated for five *C. neoformans* serotype A strains (*cap59*Δ, H99, KN99α, Mu-1, and 24064) using three different growth media (YPD, SAB, or MM) at 30°C and 37°C. Laccase, urease, phospholipase activity, and melanin production were evaluated for the four *C. neoformans* serotype A strains (H99, KN99α, Mu-1, and 24064). The growth rate of three of five strains (H99, KN99α, and Mu-1) was the same in all analyzed conditions, with those strains reaching their plateau (growth stationary phase) in less than 24 h of incubation at 30°C and 37°C (Fig. 3A through F). The strains c*ap59*Δ and 24064 demonstrated the slowest growth, requiring more than 24 h of incubation (Fig. 3A through F) to reach their plateau (growth stationary phase). All strains had laccase activity (Fig. 3G) as measured by the oxidation of ABTS. Strain 24064 had the weakest laccase activity, whereas strains H99, KN99α, and Mu-1 had comparable activity. Two laccase-deficient strains (H99 *lac1*Δ and KN99α *lac1*Δ) were used as negative controls. All strains (H99, KN99α, Mu-1, and 24064) were positive for urease production (Fig. 3H) after 72 h, as visualized by the change in the media from yellow to pink. Strains 24064 and Mu-1 had the lowest and highest urease activity, respectively. Two urease-deficient strains (H99 *ure1*Δ and KN99α *ure1*Δ) were used as negative controls. All strains had a positive phospholipase (Fig. 3I) activity, observed by the formation of a precipitation halo. Overall, all four strains had a similar precipitation index, with Mu-1 showing a higher precipitation index than H99 and KN99α, but no differences were observed between strains Mu-1 and 24064 or between H99, KN99α, and 24064. All strains produced melanin (Fig. 3J; Fig. S4), with the strains H99, KN99α, and Mu-1 being darker than 24064 as demonstrated by the higher mean gray value of the 24064 strain in comparison to the other strains (Fig. 3J).

### Re-encapsulation experiments

**Fig 4:**
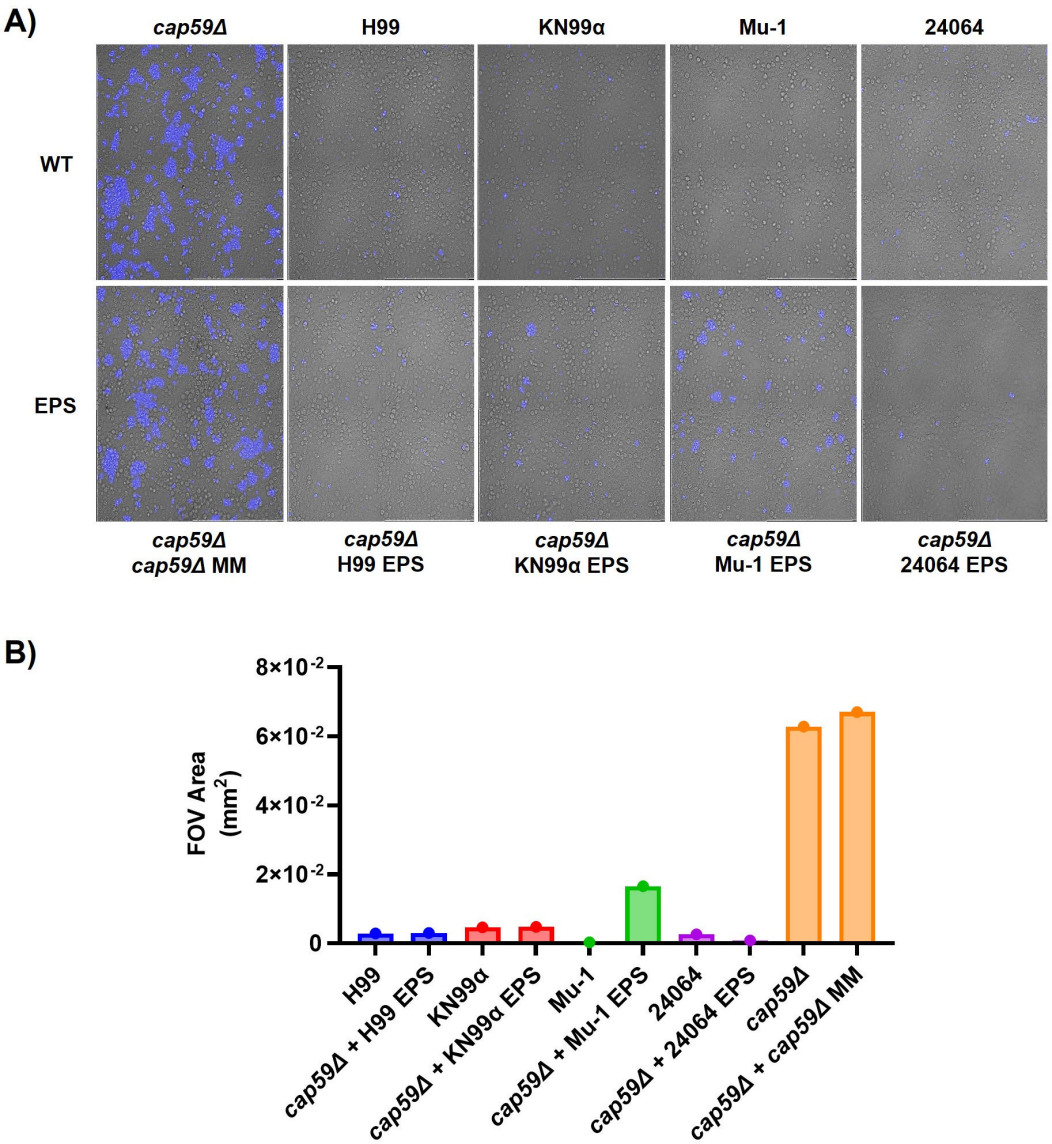
Capsular defective (acapsular) strain *cap59*∆ re-encapsulation. The re-encapsulation was performed by incubating the *cap59*Δ strains with the EPS from the H99, KN99α, Mu-1, or 24064 serotype A strains of *C. neoformans* at room temperature (24°C) for 1 h under rotation. (**A**) Phagocytosis assay showing that the re-encapsulation is preventing the acapsular strain (*cap59*∆) from being phagocytized by J774 macrophages after 2 h of interaction when compared with their EPS donor counterpart (H99, KN99α, Mu-1, or 24064). (**B**) The FOV (mathematical representation of the phagocytosis) area occupied by the fluorescent (Uvitex 2B) *C. neoformans* strains. The images represent the sum of nine squares (3 × 3) with 0.1 mm^2^ area each (0.9 mm^2^ total area), 40× magnification. Non-phagocytosed yeast was removed by the washing steps before fixation.

Notably, when a shed polysaccharide is added to acapsular strains, it attaches to the cell surface, creating a new proto capsule. This new capsular layer can interfere with phagocytosis by preventing macrophage receptors from recognizing the fungal cell. This property allows the comparison of certain biological properties of shed polysaccharides in the context of acquired characteristics for the acapsular cell. Consequently, we evaluated the anti-phagocytic capacity of shed polysaccharide from the four serotype A strains by adding it to a non-encapsulated strain and studying its interaction with macrophages. Re-encapsulation was confirmed by immunofluorescence (Fig. S6), such that the *cap59*∆ strain incubated with its own media alone had no capsule detection. In contrast, the incubation with media supernatant from strains H99, KN99α, Mu-1, and 24064 had a positive fluorescence signal. No differences were observed for the interaction of the re-encapsulated cells with J774 macrophages (Fig. 4A). The use of an exogenous source of EPS promoted a similar macrophage/*C. neoformans* interaction when we compared the WT cells with their *cap59*∆ EPS counterpart (Fig. 4B).

### EPS effects on macrophage-like cells *in vitro*

**Fig 5:**
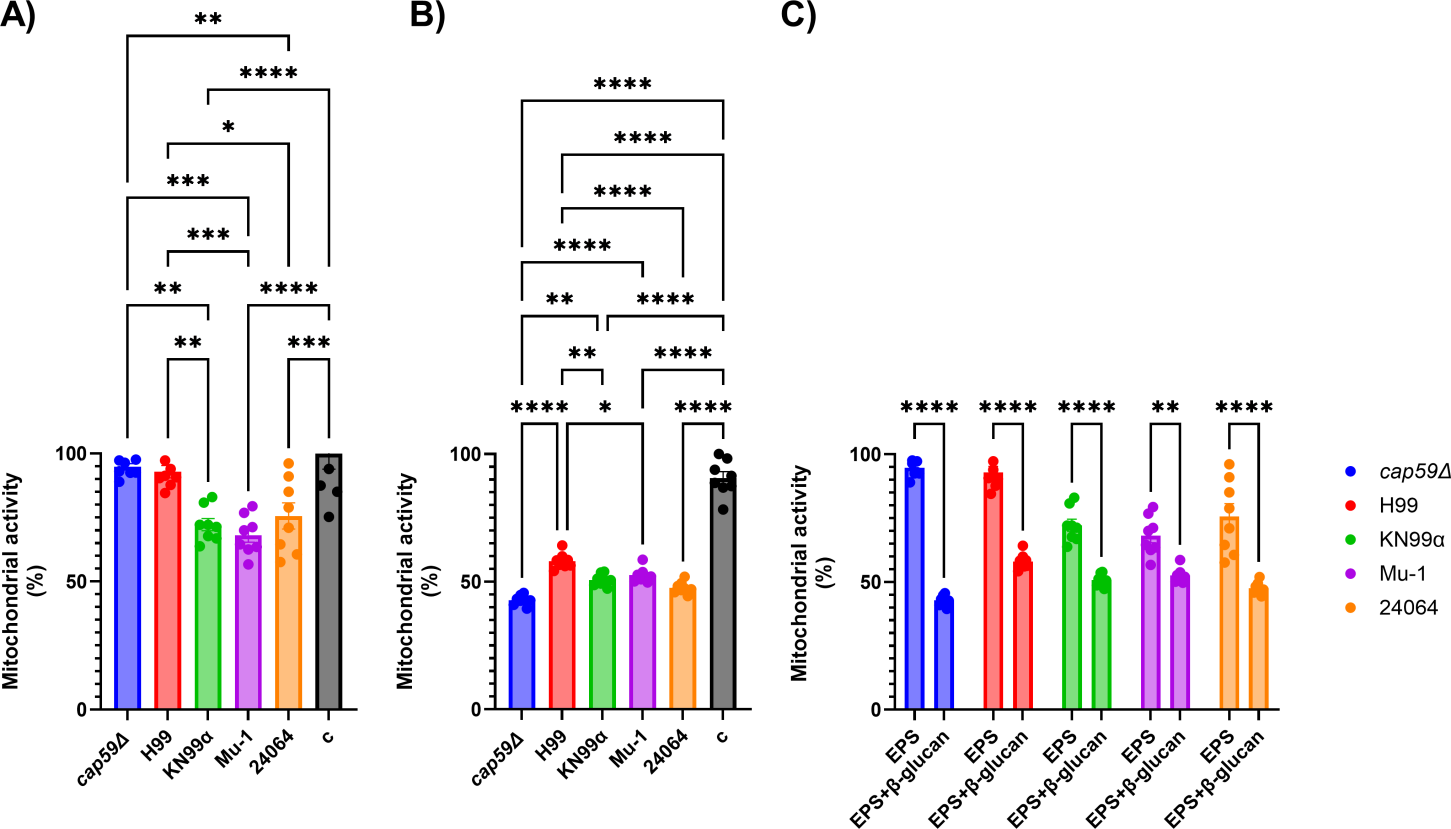
Mitochondrial activity of J774 macrophages after incubation with EPS from different serotype A strains of *C. neoformans* (*cap59*∆, H99, KN99α, Mu-1, or 24064) or EPS + β-glucans (Zymosan A). Mitochondrial activity was measured by the formation of formazan after the addition and incubation of MTT [3-(4,5-dimethylthiazol-2-yl)−2,5-diphenyltetrazolium bromide] for 2 h. (**A**) Mitochondrial activity of J774 cells incubated with EPS from *cap59*∆, H99, KN99α, Mu-1, or 24064, showing that the EPS from KN99α, Mu-1, or 24064 compromised the function of the macrophage mitochondria. Statistical analyses also showed that the influence of the EPS on the mitochondria varied depending on the strain. Control cells (c) incubated with cell culture media only (*n* = 8 per group). (**B**) Mitochondrial activity of J774 cells incubated with EPS from *cap59*∆, H99, KN99α, Mu-1, or 24064 + β-glucans, showing that the EPS from all analyzed strains + β-glucans compromised the function of the macrophage mitochondria. Statistical analyses also showed that the influence of the EPS + β-glucans in the mitochondria varied, depending on the strain. Control cells (c) incubated with cell culture media + β-glucans (*n* = 8 per group). (**C**) Grouped analysis comparing the difference in the mitochondrial activity between the cells incubated with only EPS from *cap59*∆, H99, KN99α, Mu-1, or 24064 and EPS from *cap59*∆, H99, KN99α, Mu-1, or 24064 + β-glucans (*n* = 8 per group). * = *P* < 0.05, ** = *P* < 0.01, *** = *P* < 0.001, **** = *P* < 0.0001 (*t*-test and or ANOVA). Multiple comparisons were corrected by Šídák’s multiple comparisons test.

Mitochondrial activity of J774 macrophage-like cells (Fig. 5) was measured after incubation with EPS from different serotype A strains of *C. neoformans* (*cap59*∆*,* H99, KN99α, Mu-1, or 24064) or EPS + β-glucans (Zymosan A). Mitochondrial activity of J774 cells incubated with EPS from *cap59*∆*,* H99, KN99α, Mu-1, or 24064 showed that the EPS from KN99α, Mu-1, or 24064 compromised the function of the macrophages' mitochondria, and statistical analyses also showed that the influence of the EPS on the mitochondria varied depending on the strain (Fig. 5A). When the β-glucans were added, the level of mitochondrial activity was reduced more drastically than observed with EPS alone (Fig. 5B and C).

**Fig 6:**
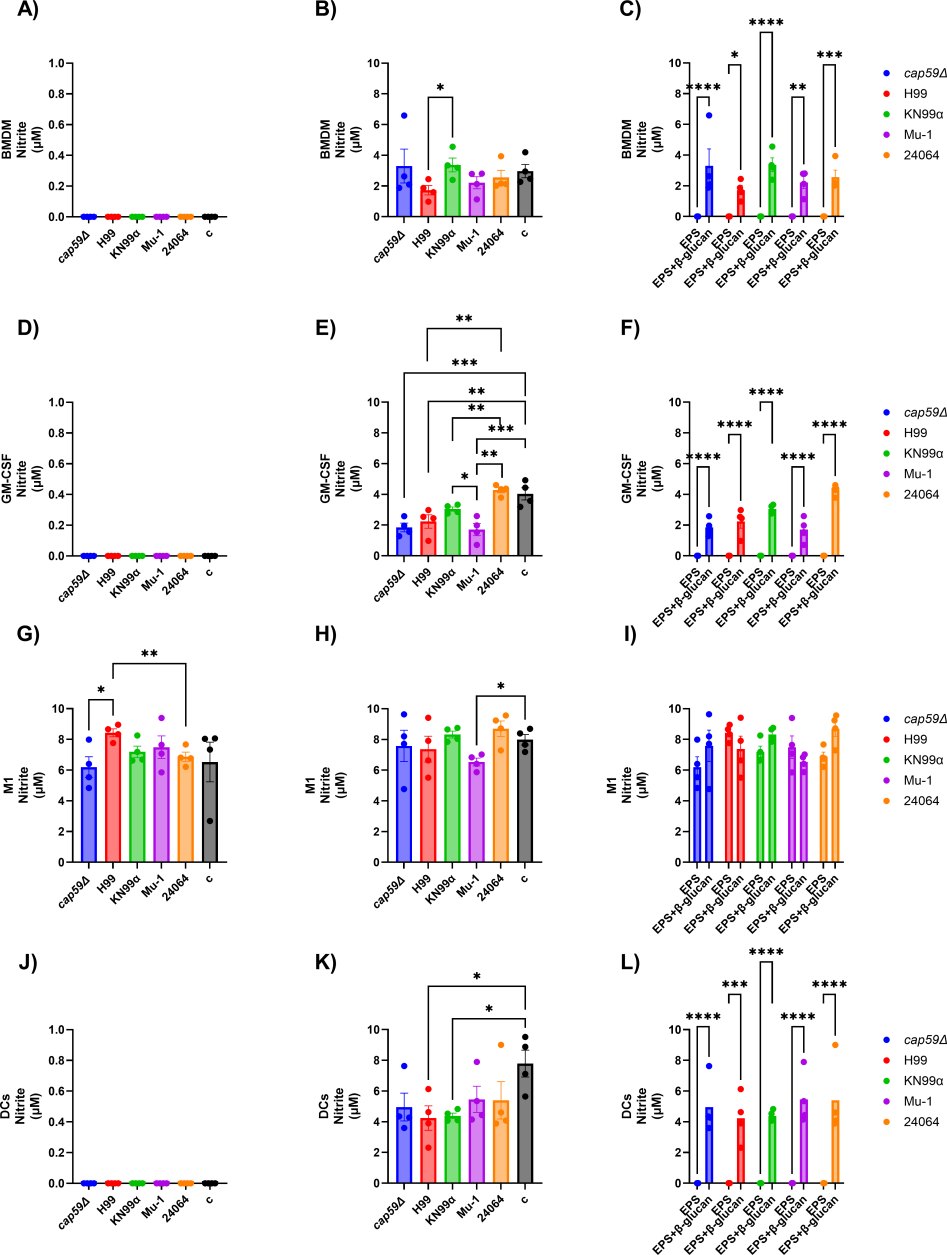
Nitrite production by bone marrow-derived cells, macrophages (BMDM, GM-CSF, and M1), or dendritic cells (DCs), after incubation with EPS from different serotype A strains of *C. neoformans* (*cap59*∆, H99, KN99α, Mu-1, or 24064) or EPS + β-glucans (Zymosan A). Nitrite was measured after 12 h of incubation. BMDM = M0 macrophages (no activation or differentiation), GM-CSF = M0 macrophages stimulated with 20 ng/mL of GM-CSF overnight, M1 = M1 macrophages activated and differentiated overnight with 100 U/mL of IFN-γ and 500 ng/mL of LPS. (**A**) Nitrite production by BMDM cells after incubation with EPS from *cap59*∆, H99, KN99α, Mu-1, or 24064 shows no nitrite production. Control cells (c) were incubated with cell culture media only in all panels (*n* = 4 per group). (**B**) Nitrite production by BMDM cells after incubation with EPS from *cap59*∆, H99, KN99α, Mu-1, or 24064 + β-glucans. The EPS from the KN99α strain elicited nitrite production compared with the H99 EPS. Control cells (c) incubated with cell culture media + β-glucans (*n* = 4 per group). (**C**) Grouped analysis comparing the difference in nitrite production of the BMDM cells incubated with only EPS from *cap59*∆, H99, KN99α, Mu-1, or 24064 and EPS from *cap59*∆, H99, KN99α, Mu-1 + β-glucans. The incubation of the cells with EPS from *cap59*∆, H99, KN99α, Mu-1, or 24064 + β-glucans increased the nitrite production when compared with the cells incubated with only EPS from *cap59*∆, H99, KN99α, Mu-1, or 24064 (*n* = 4 per group). (**D**) Nitrite production by GM-CSF-stimulated cells after incubation with EPS from *cap59*∆, H99, KN99α, Mu-1, or 24064 showed no nitrite production. Control cells (c) were incubated with cell culture media only (*n* = 4 per group). (**E**) Nitrite production by GM-CSF-stimulated cells after incubation with EPS from *cap59*∆, H99, KN99α, Mu-1, or 24064 + β-glucans. Statistical analyses showed that the influence of the EPS + β-glucans on the nitrite production also varied depending on the strain. Control cells (c) were incubated with cell culture media + β-glucans (*n* = 4 per group). (**F**) Grouped analysis comparing the difference in nitrite production of the GM-CSF stimulated cells incubated with only EPS from *cap59*∆, H99, KN99α, Mu-1, or 24064 and EPS from *cap59*∆, H99, KN99α, Mu-1, or 24064 + β-glucans. The incubation of the cells with EPS from *cap59*∆, H99, KN99α, Mu-1, or 24064 + β-glucans increased the nitrite production when compared with the cells incubated with only EPS from *cap59*∆, H99, KN99α, Mu-1, or 24064 (*n* = 4 per group). (**G**) Nitrite production by M1 cells after incubation with EPS from *cap59*∆, H99, KN99α, Mu-1, or 24064. Cells treated with *cap59*∆ and 24064 EPS had lower nitrite production levels than those treated with H99 EPS. Control cells (c) incubated with cell culture media only (*n* = 4 per group). (**H**) Nitrite production by M1 cells after incubation with EPS from *cap59*∆, H99, KN99α, Mu-1, or 24064 + β-glucans. Statistical analyses showed that the Mu-1 EPS + β-glucans had lower nitrite production levels when compared with the control cells. Control cells (c) incubated with cell culture media + β-glucans (*n* = 4 per group). (**I**) Grouped analysis comparing the difference in nitrite production of the M1 cells incubated with only EPS from *cap59*∆, H99, KN99α, Mu-1, or 24064 and EPS from *cap59*∆, H99, KN99α, Mu-1 + β-glucans (*n* = 4 per group). (**J**) Nitrite production by DC cells after incubation with EPS from *cap59*∆, H99, KN99α, Mu-1, or 24064 showing no nitrite production. Control cells (c) incubated with cell culture media only (*n* = 4 per group). (**K**) Nitrite production by DC cells after incubation with EPS from *cap59*∆, H99, KN99α, Mu-1, or 24064 + β-glucans. Statistical analyses showed that the incubation of the DC cells with EPS from H99, KN99α + β-glucans had lower nitrite production compared with the control cells. Control cells (c) incubated with cell culture media + β-glucans (*n* = 4 per group). (**L**) Grouped analysis comparing the difference in nitrite production of the DC cells incubated with only EPS from *cap59*∆, H99, KN99α, Mu-1, or 24064 and EPS from *cap59*∆, H99, KN99α, Mu-1 + β-glucans. The incubation of the cells with EPS from *cap59*∆, H99, KN99α, Mu-1, or 24064 + β-glucans increased the nitrite production when compared with the cells incubated with only EPS from *cap59*∆, H99, KN99α, Mu-1, or 24064 (*n* = 4 per group). * = *P* < 0.05, ** = *P* < 0.01, *** = *P* < 0.001, **** = *P* < 0.0001 (*t*-test and or ANOVA). Multiple comparisons were corrected by Šídák’s multiple comparisons test.

The nitrite production of bone marrow-derived cells, macrophages (BMDM, GM-CSF, and M1), or dendritic cells (DCs), after incubation with EPS from different serotype A strains of *C. neoformans* (*cap59*∆, H99, KN99α, Mu-1, or 24064) or EPS + β-glucans (Zymosan A) varied based on the type of cell utilized, their activation status, and the EPS strain type (Fig. 6A through L; Fig. S7; Table S3 to S6). The analysis of the interaction of the dendritic cells with the different types of EPS and the different types of EPS + β-glucans demonstrated differences in the nitrite production level based on the EPS strain type origin.

**Fig 7:**
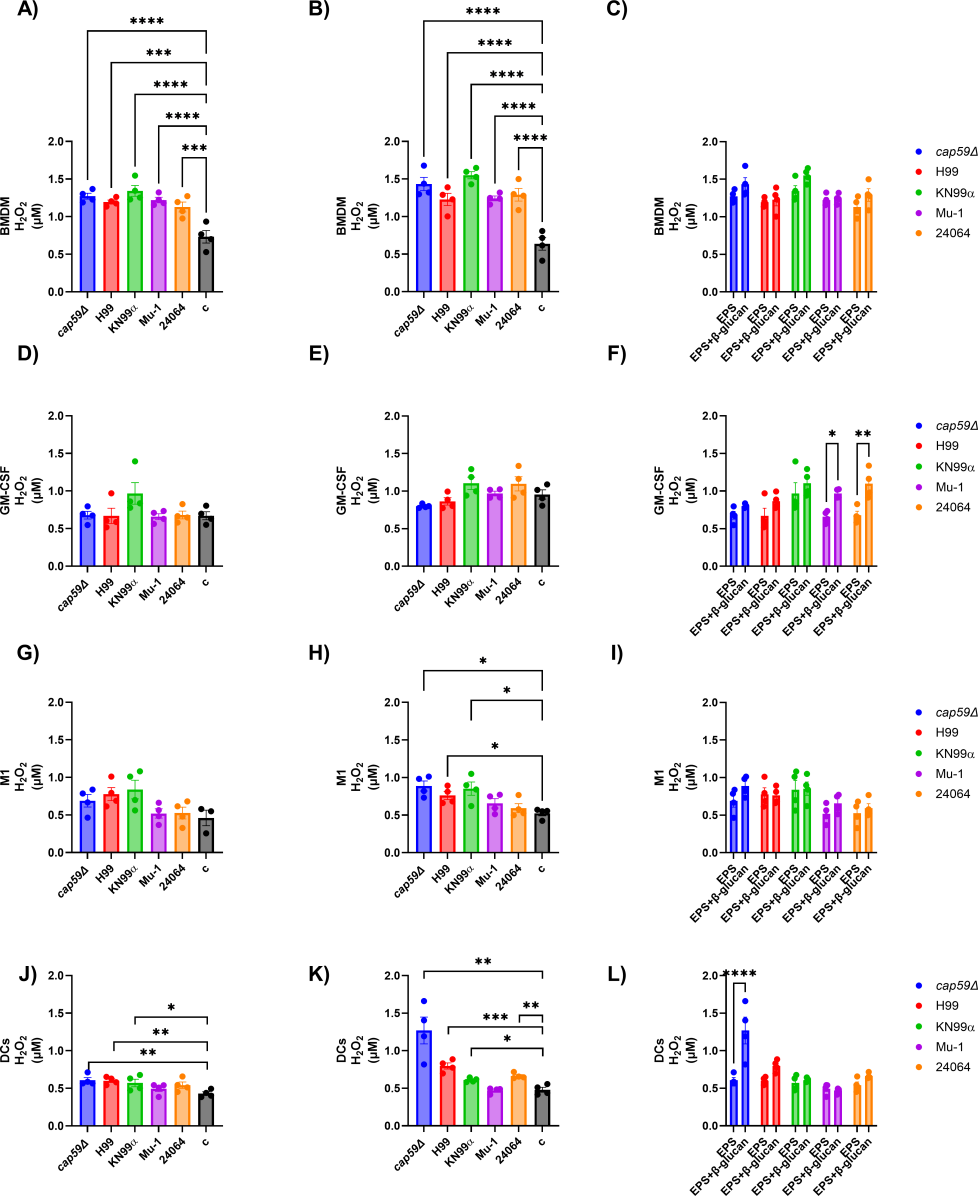
ROS production by bone marrow-derived cells, macrophages (BMDM, GM-CSF, and M1), or dendritic cells (DCs), after incubation with EPS from different serotype A strains of *C. neoformans* (*cap59*∆, H99, KN99α, Mu-1, or 24064) or EPS + β-glucans (Zymosan A). ROS was measured after 12 h of incubation. BMDM = M0 macrophages (no activation or differentiation), GM-CSF = M0 macrophages stimulated with 20 ng/mL of GM-CSF overnight, M1 = M1 macrophages activated and differentiated overnight with 100 U/mL of IFN-γ and 500 ng/mL of LPS. (**A**) ROS production by BMDM cells after incubation with EPS from *cap59*∆, H99, KN99α, Mu-1, or 24064. Statistical analyses showed that the control cells had the lowest levels of ROS production. Control cells (c) incubated with cell culture media only (*n* = 4 per group). (**B**) ROS production by BMDM cells after incubation with EPS from *cap59*∆, H99, KN99α, Mu-1, or 24064 + β-glucans. Statistical analyses showed that the control cells had the lowest levels of ROS production. Control cells (c) incubated with cell culture media + β-glucans (*n* = 4 per group). (**C**) Grouped analysis comparing the difference in ROS production of the BMDM cells incubated with only EPS from *cap59*∆, H99, KN99α, Mu-1, or 24064 and EPS from *cap59*∆, H99, KN99α, Mu-1 + β-glucans (*n* = 4 per group). (**D**) ROS production by GM-CSF-stimulated cells after incubation with EPS from *cap59*∆, H99, KN99α, Mu-1, or 24064. Control cells (c) incubated with cell culture media only (*n* = 4 per group). (**E**) ROS production by GM-CSF-stimulated cells after incubation with EPS from *cap59*∆, H99, KN99α, Mu-1, or 24064 + β-glucans. Control cells (c) incubated with cell culture media + β-glucans (*n* = 4 per group). (**F**) Grouped analysis comparing the difference in ROS production of the GM-CSF stimulated cells incubated with only EPS from *cap59*∆, H99, KN99α, Mu-1, or 24064 and EPS from *cap59*∆, H99, KN99α, Mu-1, or 24064 + β-glucans. Statistical analyses showed that Mu-1 and 24064 EPS had an increase in ROS production with the addition of + β-glucans (*n* = 4 per group). (**G**) ROS production by M1 cells after incubation with EPS from *cap59*∆, H99, KN99α, Mu-1, or 24064. Control cells (c) incubated with cell culture media only (*n* = 4 per group). (**H**) ROS production by M1 cells after incubation with EPS from *cap59*∆, H99, KN99α, Mu-1, or 24064 + β-glucans. Statistical analyses showed that the *cap59*∆, H99, and KN99α EPS + β-glucans had higher ROS production levels when compared with the control cells. Control cells (c) incubated with cell culture media + β-glucans (*n* = 4 per group). (**I**) Grouped analysis comparing the difference in ROS production of the M1 cells incubated with only EPS from *cap59*∆, H99, KN99α, Mu-1, or 24064 and EPS from *cap59*∆, H99, KN99α, Mu-1 + β-glucans (*n* = 4 per group). (**J**) ROS production by DC cells after incubation with EPS from *cap59*∆, H99, KN99α, Mu-1, or 24064. Statistical analyses showed that *cap59*Δ, H99, or KN99α had higher ROS production levels when compared with the control cells. Control cells (c) incubated with cell culture media only (*n* = 4 per group). (**K**) ROS production by DC cells after incubation with EPS from *cap59*∆, H99, KN99α, Mu-1, or 24064 + β-glucans. Statistical analyses showed that the incubation of the DC cells with EPS from *cap59*∆, H99, KN99α, or 24064 + β-glucans had higher ROS production levels when compared with the control cells. Control cells (c) incubated with cell culture media + β-glucans (*n* = 4 per group). (**L**) Grouped analysis comparing the difference in ROS production of the DC cells incubated with only EPS from *cap59*∆, H99, KN99α, Mu-1, or 24064 and EPS from *cap59*∆, H99, KN99α, or Mu-1 + β-glucans. The incubation of the cells with EPS from *cap59*∆ + β-glucans increased the ROS production when compared with the cells incubated with only EPS from *cap59*∆ (*n* = 4 per group). * = *P* < 0.05, ** = *P* < 0.01, *** = *P* < 0.001, **** = *P* < 0.0001 (*t*-test and/or ANOVA). Multiple comparisons were corrected by Šídák’s multiple comparisons test.

The reactive oxygen species (ROS) production of bone marrow-derived cells, macrophages (BMDM, GM-CSF, and M1), or dendritic cells (DCs), after incubation with EPS from different serotype A strains of *C. neoformans* (*cap59*∆, H99, KN99α, Mu-1, or 24064) or EPS + β-glucans (Zymosan A) varied based on the type of cell utilized, their activation status, and the EPS strain type (Fig. 7A through L; Fig. S7; Table S3 to S6). The analysis of the interaction of the M1 macrophages or dendritic cells with the different types of EPS, with and without β-glucans, demonstrated differences in the ROS production level based on the EPS strain type origin.

### Virulence of serotype A strains in mice

**Fig 8:**
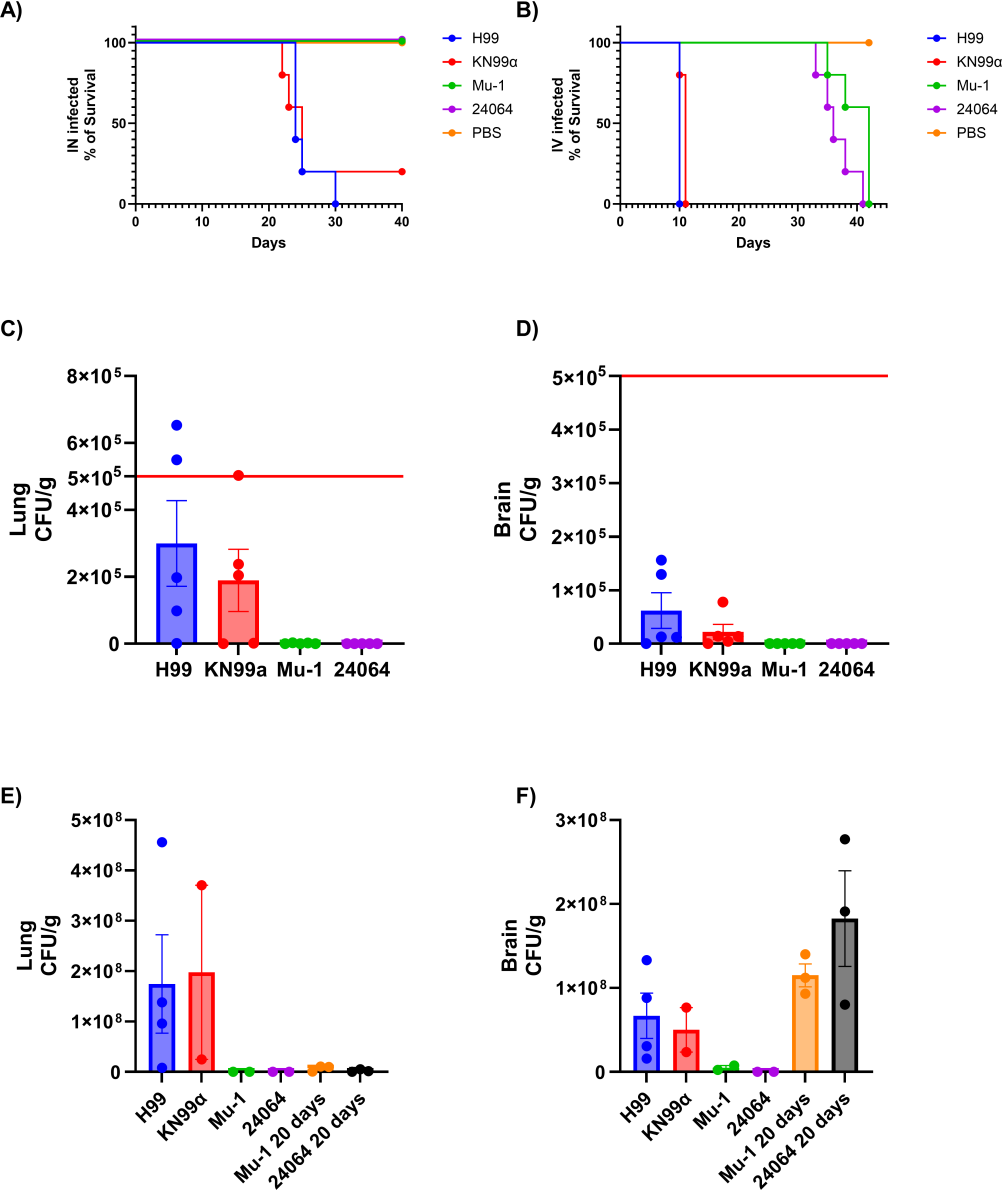
Survival and fungal burden studies (CFU). Mice were infected with *C. neoformans* strains H99, KN99α, Mu-1, or 24064, either by intranasal (IN) route or intravenous (IV) route, with 5 × 10^5^ yeast suspended in 20 µL of PBS (10 µL per nostril) or 5 × 10^5^ yeast in 100 µL of PBS, respectively. Mice were euthanized 5 or 20 days after infection. (**A**) Survival of IN-infected mice (*n* = 5 per group). (**B**) Survival of IV-infected mice (*n* = 5 per group). (**C**) Lung CFU of IN-infected mice (*n* = 5 per group). (**D**) Brain CFU of IN-infected mice (*n* = 5 per group). (**E**) Lung CFU in IV-infected mice after 20 days of infection (*n* = 4 for the H99 group, *n* = 2 for the KN99α group, *n* = 2 for the Mu-1 and 24064 groups, and *n* = 3 for the Mu-1 [20 days] and 24064 [20 days]). (**F**) Brain CFU of IV-infected mice after 20 days of infection (*n* = 4 for the H99 group, *n* = 2 for the KN99α group, *n* = 2 for the Mu-1 and 24064 groups, and *n* = 3 for the Mu-1 [20 days] and 24064 [20 days]). The red line represents the original inoculum (5 × 10^5^). For the IV infection, dead animals from the H99 (1) or KN99α (3) group had similar CFU counts but were removed from the final analysis because they died 12 h before euthanasia for CFU determination. Data represent the second independent replicate.

The survival studies showed that when infected by the IN route (Fig. 8A), mice had a mean survival time of 20 days when infected with the multi-motif strains H99 or KN99α. No death occurred during infection with the single-motif strains Mu-1 or 24064 during the duration of the experiment (40 days). Survival of the IV-infected mice (Fig. 8B) showed a mean survival time of 9 days with multi-motif strains H99 or KN99α and 35 days with single-motif strains Mu-1 or 24064. For the IN infection, a high colony-forming unit (CFU) count was observed for the groups infected with H99 or KN99α in the lungs (Fig. 8C) and brains (Fig. 8D) after 20 days of infection. No CFUs or very few were observed in the lungs and brains for the groups infected with Mu-1 or 24064 after 20 days of IN infection. Based on the results of the survival studies, the infection times were modified for the IV-infected mice to compare the levels of pathogenicity in the most advanced stage of the infection, but prior to the death of the mice. For the IV-infected mice, a high CFU count was observed for the groups infected with H99 or KN99α in the lungs (Fig. 8E) and brains (Fig. 8F) after 5 days of infection. No or very few CFU were observed in the lungs and brains for the groups infected with Mu-1 or 24064 after 5 days of IV infection. After 20 days of IV infection, no or very few CFU were observed in the lungs (Fig. 8E) for the groups Mu-1 20 days and 24064 20 days, but a high fungal burden was observed in the brains (Fig. 8F) of the same group. Hence, strains H99 or KN99α were consistently more virulent than Mu-1 and 24064 in mice infected by different routes.

### Host response to intranasal (IN) serotype A strain-infected mice

**Fig 9:**
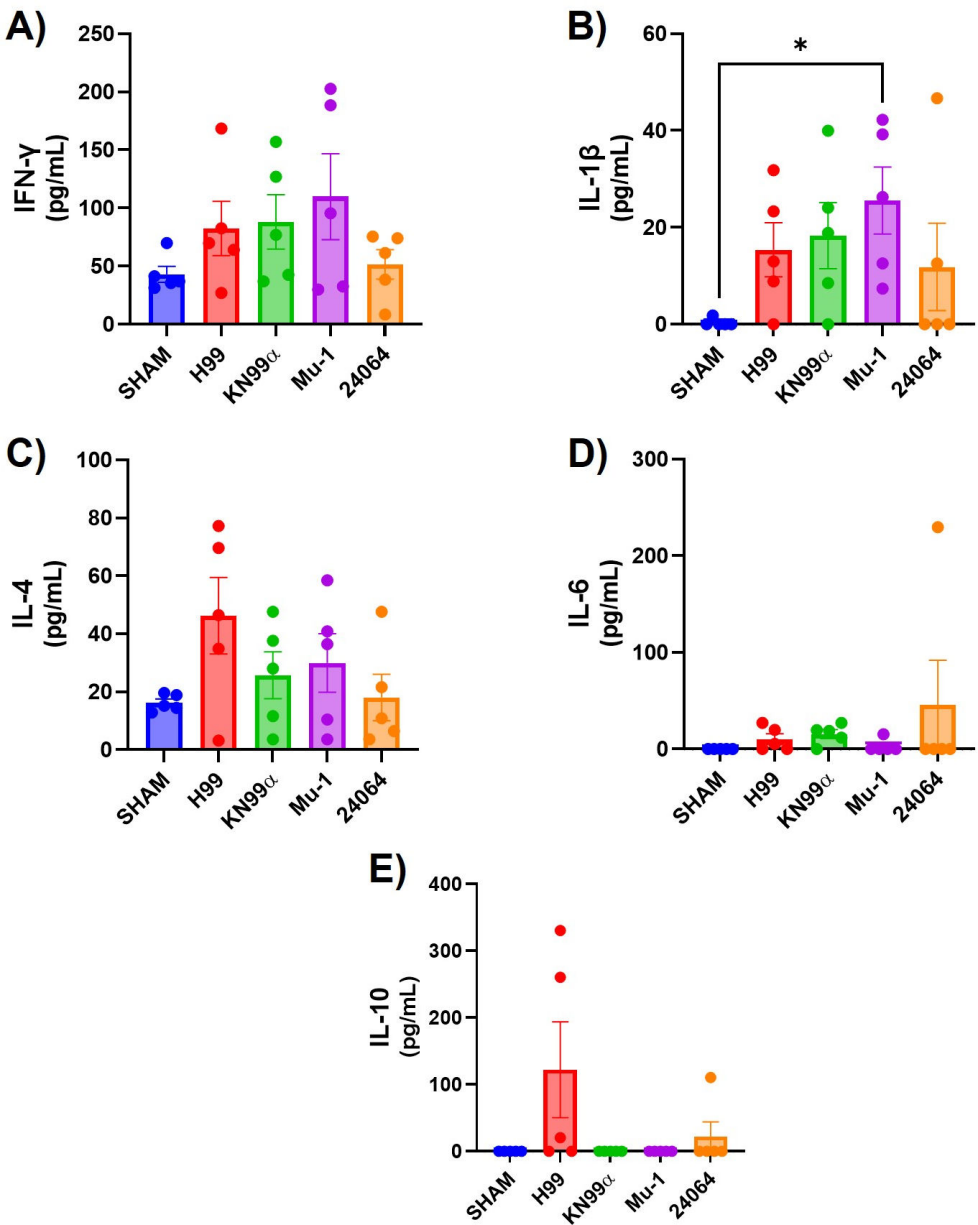
IN-infected mice lungs, cytokines after 20 days of infection. (**A**) IFN-γ (*n* = 5 per group). (**B**) IL-1β (*n* = 5 per group). (**C**) IL-4 (*n* = 5 per group). (**D**) IL-6 (*n* = 5 per group). (**E**) IL-10 (*n* = 5 per group). All infected lungs had higher levels of IFN-γ, IL-1β, and IL-4 compared with the lungs from non-infected mice (SHAM). * = *P* < 0.05 (*t*-test and or ANOVA). Multiple comparisons were corrected by Šídák’s multiple comparisons test.

**Fig 10:**
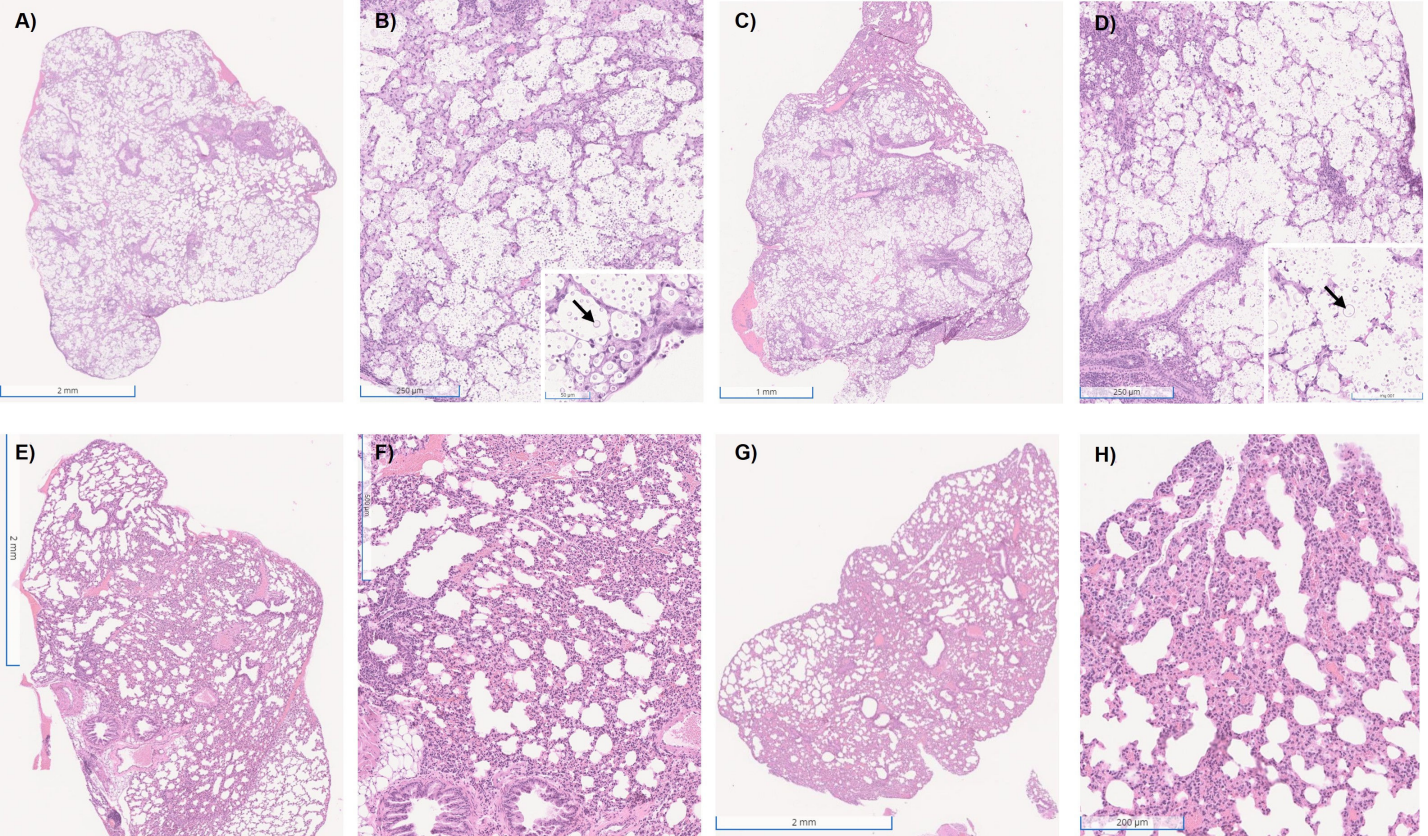
Histological findings after 20 days of IN infection. After euthanasia, randomly selected lungs had a small tissue sample piece aseptically excised and preserved in formalin until analysis. (**A**) H99-infected mice 1× magnification. (**B**) H99-infected mice 6× magnification; insert box 20× magnification and black arrow showing a cryptococcal cell. (**C**) KN99α 1× magnification. (**D**) KN99α 6× magnification; insert box 20× magnification and black arrow showing a cryptococcal cell. (**E**) Mu-1 infected mice 1× magnification. (**F**) Mu-1 infected mice 6× magnification. (**G**) 24064 infected mice 1× magnification. (**H**) 24064 infected mice 6× magnification. HE stains.

**Fig 11:**
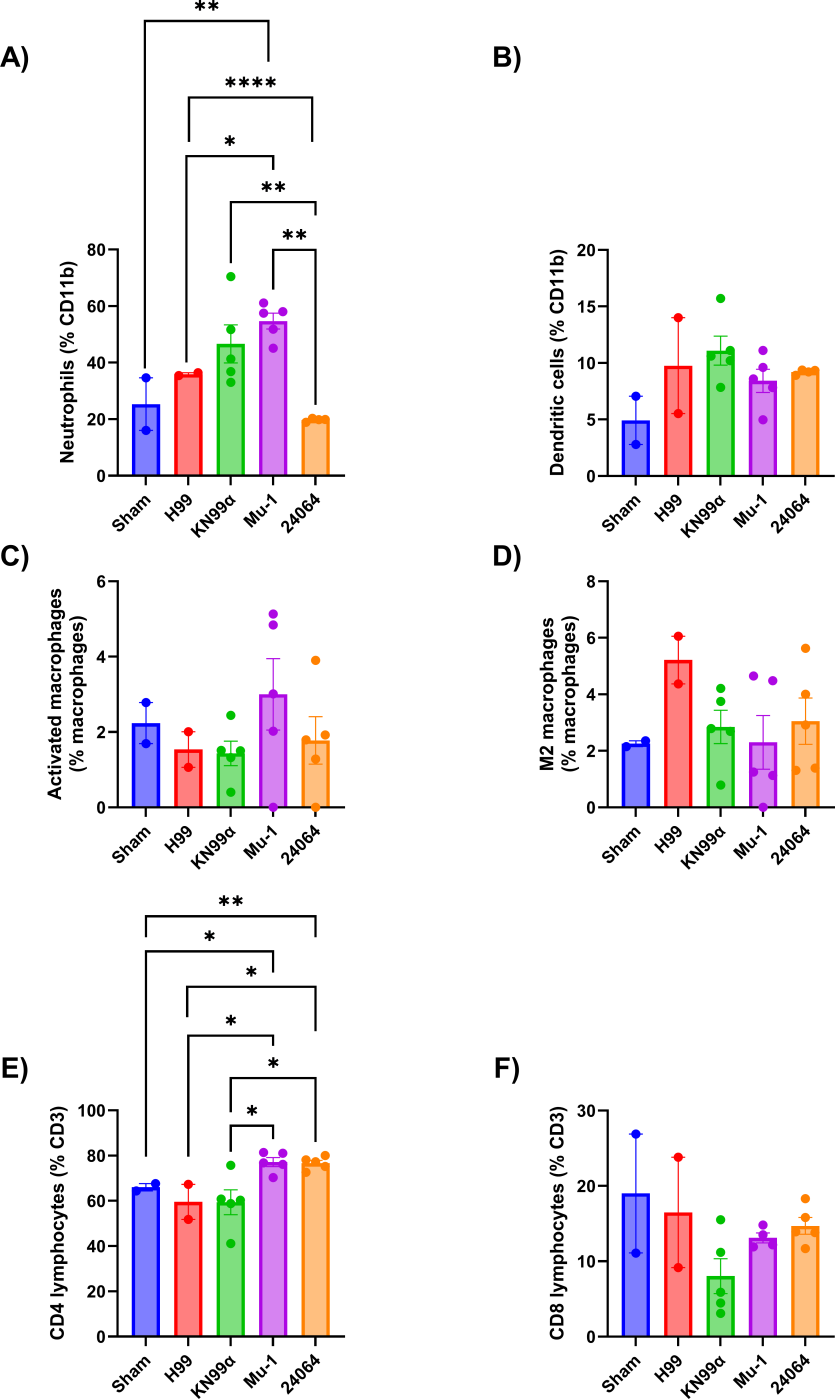
Flow cytometry of the lung of IN-infected mice after 20 days of infection. (**A**) Neutrophils (*n* = 2 for the sham group, *n* = 2 for the H99 group, *n* = 4 for the KN99α group, *n* = 5 for the Mu-1 and 24064). (**B**) Dendritic cells (*n* = 2 for the sham group, *n* = 2 for the H99 group, *n* = 4 for the KN99α group, *n* = 5 for the Mu-1 and 24064). (**C**) Activated macrophages (*n* = 2 for the sham group, *n* = 2 for the H99 group, *n* = 4 for the KN99α group, *n* = 5 for the Mu-1 and 24064). (**D**) M2 macrophages (*n* = 2 for the sham group, *n* = 2 for the H99 group, *n* = 4 for the KN99α group, *n* = 5 for the Mu-1 and 24064). (**E**) CD4+ T lymphocytes (*n* = 2 for the sham group, *n* = 2 for the H99 group, *n* = 4 for the KN99α group, *n* = 5 for the Mu-1 and 24064). (**F**) CD8+ T lymphocytes (*n* = 2 for the sham group, *n* = 2 for the H99 group, *n* = 4 for the KN99α group, *n* = 5 for the Mu-1 and 24064). * = *P* < 0.05, ** = *P* < 0.01, **** = *P* < 0.0001 (*t*-test and or ANOVA). Multiple comparisons were corrected by Šídák’s multiple comparisons test.

The inflammatory response to infection with the four serotype A strains was studied by comparing organ content of specific cytokines, the composition of the cellular infiltrates, and histological examination of infected tissues. The cytokine analysis (Fig. 9A through E) evaluated 20 days post-infection showed that all strains had higher levels of IFN-γ, IL-1β, and IL-4 when compared with the non-infected mice (SHAM). The multi-motif strain H99 elicited the highest levels of IL-4 and IL-10, and the single-motif strain Mu-1 the highest levels of IFN-γ and IL-1β. For the mice IN infected with the multi-motif strains H99 or KN99α, less inflammation, similar to the SHAM group (Fig. S8), was observed, but a high presence of cryptococcal cells was observed in the lung parenchyma and alveoli (Fig. 10A through D). For mice IN infected with the single-motif strains Mu-1 or 24,064, no or few cryptococcal cells were observed, but the lung tissue showed a high presence of inflammatory infiltrated cells and enlargement of the parenchyma and alveoli walls (Fig. 10E through H). The flow cytometry analysis (Fig. 11) of the inflammatory cells from the lung of IN-infected mice after 20 days showed that the neutrophils (Fig. 11A) were higher for the H99, KN99α and Mu-1 when compared with the strain 24064. No statistical differences were observed for the number of dendritic cells (Fig. 11B). Mu-1 and 24064 had higher numbers of activated macrophages (Fig. 11C), but no statistical difference was detected. H99 had higher numbers of M2 polarized macrophages (Fig. 11D), but no statistical difference was detected. CD4+ lymphocytes (Fig. 11E) were significantly increased for the Mu-1 and 24064 strains when compared with the H99 and KN99α strains. CD8+ lymphocyte numbers were higher for the H99 strains, followed by Mu-1 and 24064 strains, but no statistical difference was detected.

### Host response to intravenous (IV) serotype A strain-infected mice

**Fig 12:**
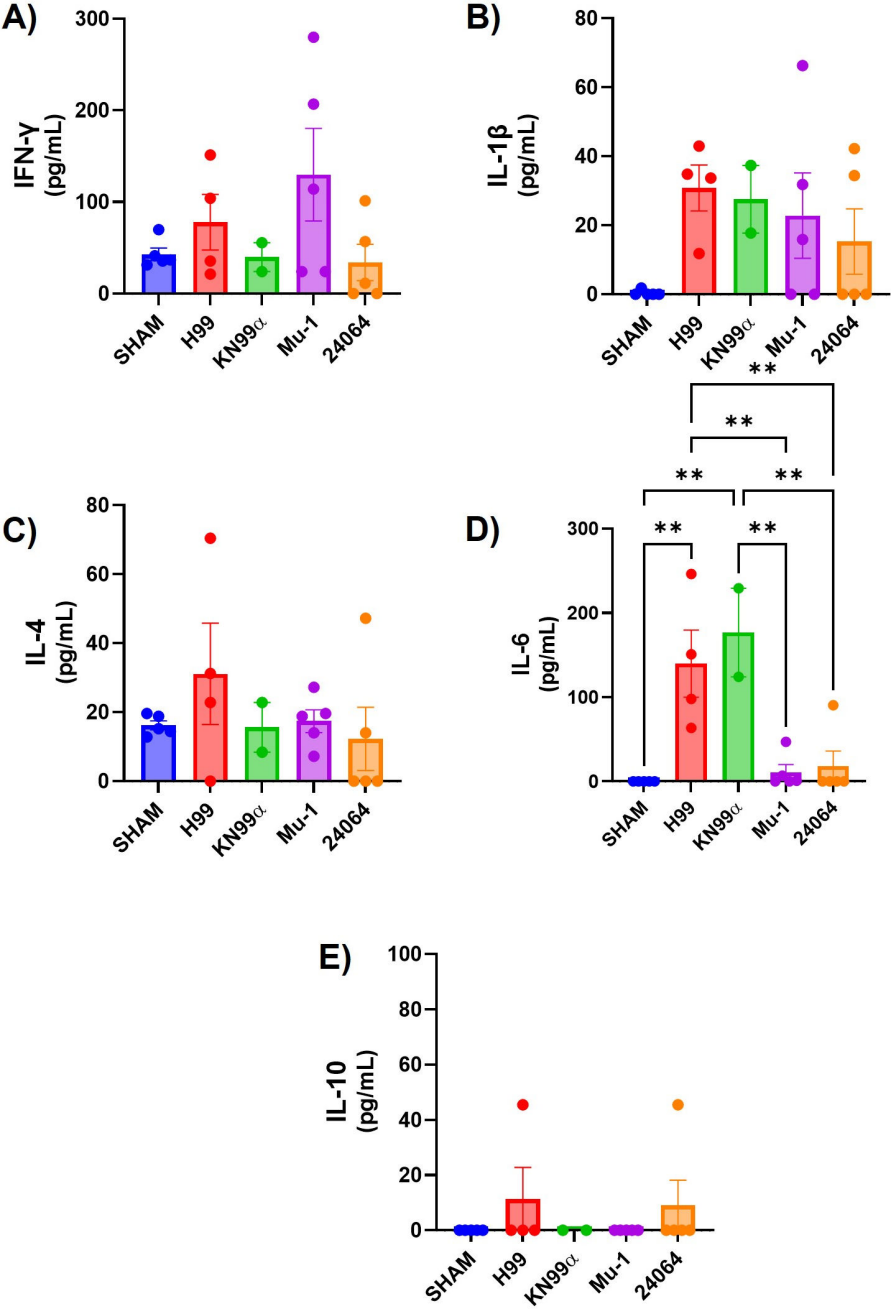
Lungs cytokines after 5 and/or 20 days of IV infection. (**A**) IFN-γ (*n* = 5 for the sham group, *n* = 4 for the H99 group, *n* = 2 for the KN99α group, *n* = 5 for the Mu-1 and 24064). (**B**) IL1-β (A) IFN-γ (*n* = 5 for the sham group, *n* = 4 for the H99 group, *n* = 2 for the KN99α group, *n* = 5 for the Mu-1 and 24064). (**C**) IL-4 (*n* = 5 for the sham group, *n* = 4 for the H99 group, *n* = 2 for the KN99α group, *n* = 5 for the Mu-1 and 24064). (**D**) IL-6 (*n* = 5 for the sham group, *n* = 4 for the H99 group, *n* = 2 for the KN99α group, *n* = 5 for the Mu-1 and 24064). (**E**) IL-10 (*n* = 5 for the sham group, *n* = 4 for the H99 group, *n* = 2 for the KN99α group, *n* = 5 for the Mu-1 and 24064). All infected lungs had higher levels of IFN-γ, IL-1β, and IL-4 when compared with the lungs from non-infected mice (SHAM). ** = *P* < 0.01 (*t*-test and or ANOVA). Multiple comparisons were corrected by Šídák’s multiple comparisons test.

**Fig 13:**
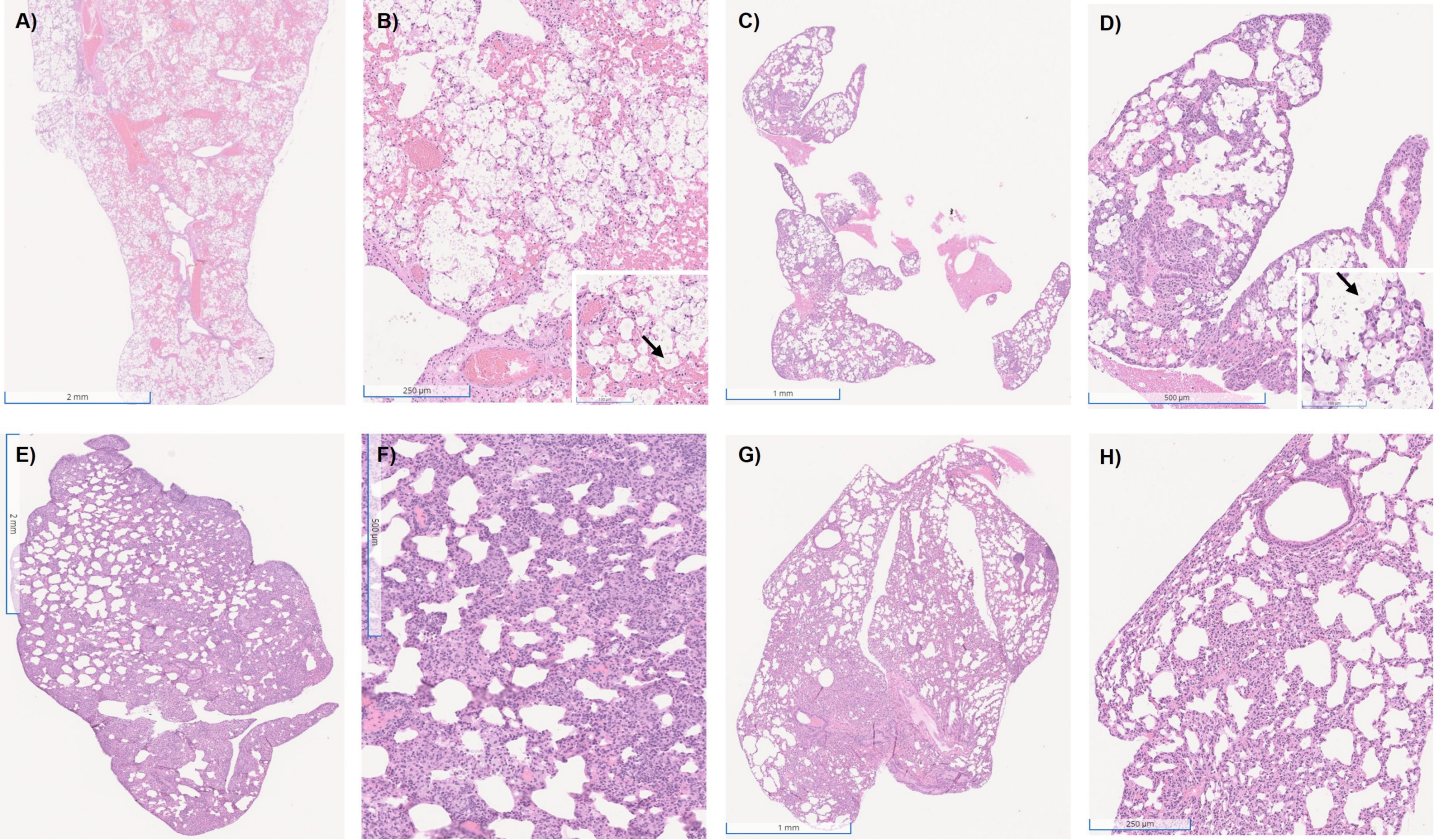
Histological findings after 5 days of IV infection. After euthanasia, randomly selected lungs had a small tissue sample piece aseptically excised and preserved in formalin until analysis. (**A**) H99-infected mice 1× magnification. (**B**) H99-infected mice 6× magnification; insert box 20× magnification and black arrow showing a cryptococcal cell. (**C**) KN99α 1× magnification. (**D**) KN99α 6× magnification; insert box 20× magnification and black arrow showing a cryptococcal cell. (**E**) Mu-1 infected mice 1× magnification. (**F**) Mu-1 infected mice 6× magnification. (**G**) 24064 infected mice 1× magnification. (**H**) 24064 infected mice 6× magnification. HE stains.

The inflammatory response to infection with the four serotype A strains was studied by organ content of specific cytokines, the composition of the cellular infiltrates, and histological examination of infected tissues. The cytokine analysis of IV-infected mice (Fig. 12A through E) after 5 days post-infection (multi-motif strains H99 or KN99α) and 20 days post-infection (Mu-1 or 24064) showed that H99 and KN99α had the highest levels of IL-6 and IL1-β, H99 having a slightly higher level of IL-4, and Mu-1 had the highest IFN-γ levels. Mice IV infected with the multi-motif strains H99 or KN99α manifested less inflammation, similar to the SHAM group (Fig. S8), but a high presence of cryptococcal cells was observed in the lung parenchyma and alveoli (Fig. 13A through D). Mice IV infected with the single-motif strains Mu-1 or 24064 had no or few observable cryptococcal cells, but the lung tissue showed a high presence of inflammatory infiltrated cells and enlargement of the parenchyma and alveoli walls (Fig. 13E through H).

### Initial immunoresponse timeframe to intratracheal (IT) serotype A strain-infected mice

**Fig 14:**
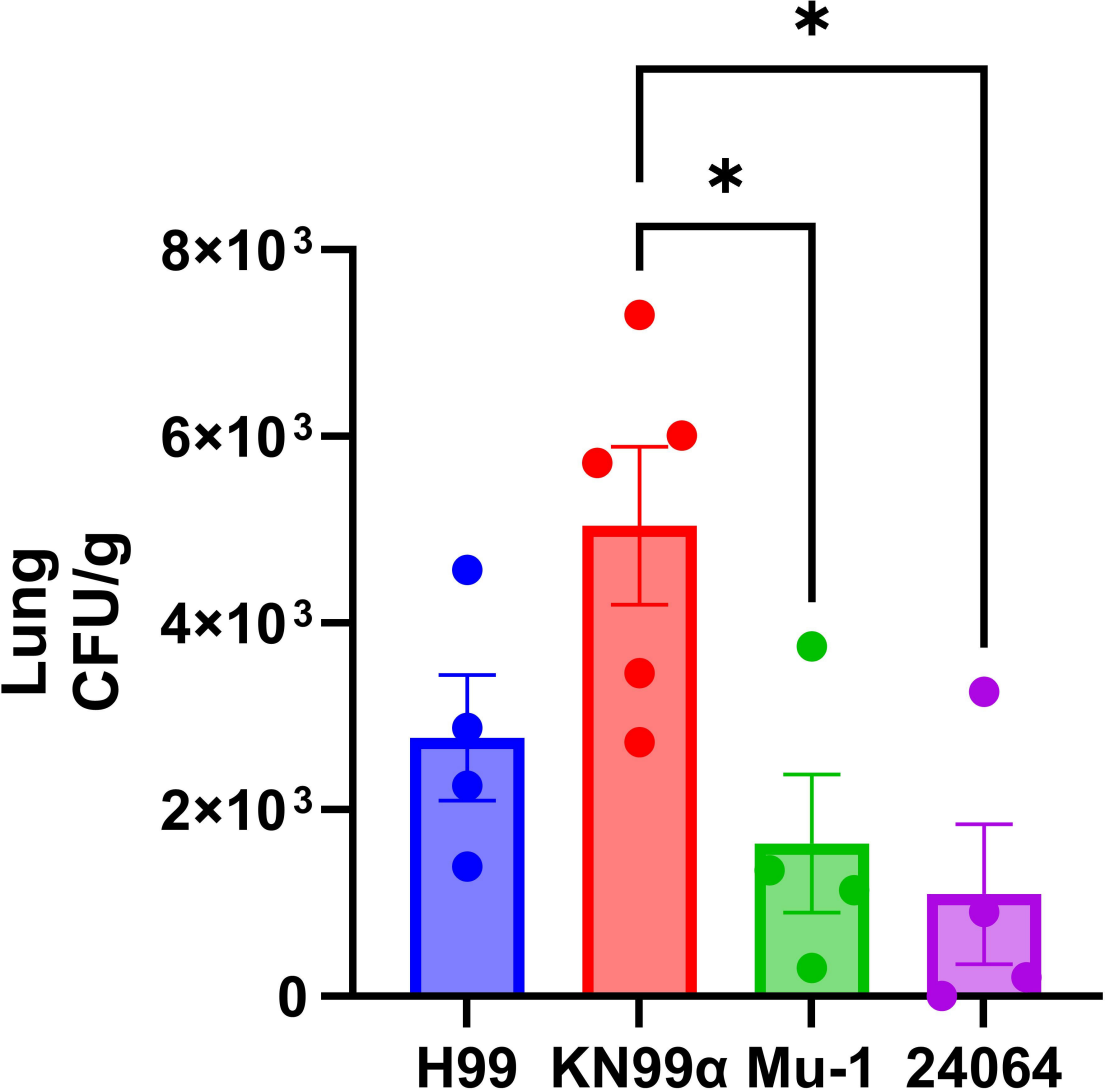
Lung fungal load (CFU) after 12 h of infection. Mice were infected with *C. neoformans* strains (H99, KN99α, Mu-1, or 24064) by intratracheal (IT) route with 5 × 10^5^ yeast suspended in 50 µL of PBS (*n* = 4 for the H99 group, *n* = 5 for the KN99α group, *n* = 4 for the Mu-1, and *n* = 4 for the 24064). The euthanasia occurred 12 h after infection. Data represent the one experiment * = *P* < 0.05. (*t*-test and or ANOVA). Multiple comparisons were corrected by Šídák’s multiple comparisons test.

The initial immunoresponse for the cryptococcal infection was studied by quantifying the lung fungal load (CFU) after 12 h of infection (Fig. 14). Mice were infected with *C. neoformans* strains (H99, KN99α, Mu-1, or 24064) by IT route, and the CFU counts showed that Mu-1 and 24064-infected mouse groups had lower CFU loads in their lungs when compared with H99- and KN99α-infected groups.

## DISCUSSION

The polysaccharide capsule is the major virulence factor of the *Cryptococcus* genus (30). The capsule’s highly complex composition and structure can modulate the immune system in different ways. The mechanism by which the capsule modulates the immune system is not yet well established. *C. neoformans* capsule can induce macrophage polarization from M0 to M2, which stimulates the release of anti-inflammatory cytokines, such as IL-4 and IL-6, along with stimulation of regulatory cytokines like IL-10 (22). It also has buffering abilities that change the pH of phagolysosomes and promote fungal survival inside phagocytic cells (25). Although we know that the capsular phenotype makes a contribution to the *C. neoformans* virulence composite, qualitative aspects of polysaccharide structural differences on virulence have not been explored. In this study, we compared for the first time the virulence and pathogenesis of four *C. neoformans* serotype A strains that differ in the complexity of their EPS structure.

Studying the contribution of compositional and structural features of capsular polysaccharides to virulence is difficult, given that the molecular machinery responsible for motif formation is not defined, and this prevents making defined mutants with or without the polysaccharide motifs of interest. Consequently, we have relied on comparative analysis of strains of the same serotype that differ in polysaccharide motif composition. One limitation of this approach is that the strains are not isogenic, and one cannot be sure that differences in virulence are solely the result of differences in polysaccharide structure. We selected two single-motif strains (Mu-1 and 24064) that had been characterized (29) and then identified the motifs of two multi-motif strains (H99 and KN99α) using 1D proton nuclear magnetic resonance (NMR). We note that H99 and KN99α differ in motif composition despite being closely related, suggesting that this feature of the capsule may not be stable. These differences in GXM composition may have arisen through microevolution, since differences in both virulence and polysaccharide were reported for the 24067 strains maintained in different labs (31). On the other hand, we noted that Mu-1 and 24064, the GXM composition was stable when compared with the original studies from the Cherniak laboratory (29).

The presence or absence of multiple motifs did not correlate with cell size or its ability to form a capsule (9, 10, 32). Additionally, different culture media conditions did not favor cell size or capsule growth in one motif strain over the other when comparing the cells in the same condition (9, 10, 32). Greater differences in cell size or capsule growth can be observed when each strain is compared in different cultivation media, as SAB media had smaller cells and capsules, whereas the cells cultivated in MM had the biggest capsule sizes (9, 10, 32). Some of the most important mechanisms of virulence and pathogenesis of *C. neoformans* are growth rate, laccase, urease, phospholipase, and melanization (9, 33). Phenotypically, no differences were found between the analyzed strains regarding these virulence factors *in vitro*, other than the differences in the capsule motif composition*.* Hence, the four serotype A strains had comparable expressions of the major characteristics associated with virulence, although small differences may still play a role in the virulence and pathogenicity *in vivo*.

Although it is currently not possible to generate isogenic strains differing in capsular polysaccharide motifs, a curiosity of the *C. neoformans* system allows one to evaluate biological characteristics of the polysaccharides using the phenomenon of re-encapsulation, whereby the addition of exopolysaccharides binds to the surface of acapsular cells to form a capsule-like structure. Hence, we evaluated whether a capsule formed with exogenous EPS from the multi-motif strains or single-motif strains would have any effect on the interaction between the *C. neoformans* cells and the mammalian cells *in vitro* and *in vivo.* We verified by immunofluorescence assays using the anti-GXM monoclonal antibody 18B7 that an acapsular strain (*cap59*∆) incubated with supernatants from each of the four serotype A strains had a small capsule (19–21, 34). This synthetic capsule in *cap59*∆ delayed the phagocytosis of the re-encapsulated yeast at a similar level to the wild-type encapsulated counterparts up to 2 h of interaction, indicating that EPS from each of the four strains inhibited phagocytosis to a comparable extent. Hence, EPS with different motif compositions was each effective at hindering phagocytosis.

In the capsule reconstitution experiments, we note that attachment of EPS to the surface of acapsular cells does not result in a native capsule. The exact mechanism of GXM binding to the cell wall to form the polysaccharide capsule is unknown; no protein or anchor has been described as responsible for capsular attachment, although chitin, chitosan, and chitin-like molecules are somehow associated with the attachment of GXM to the cell wall (35). The *cap59*∆ strain does produce GXM but fails to release it. The lack of the capsule also reduces the virulence of the *cap59*∆ strain; complementation of the CAP59 gene can restore virulence (36, 37). The transport of GXM from inside the cell to the extracellular environment occurs by extracellular vesicle release (38), and CAP59 is one of the responsible genes associated with GXM extracellular transport and vesicle release (39). Nevertheless, these experiments provide the means to compare the effects of cell-bound EPS on acapsular cells.

Given the limitations in establishing causality between GXM complexity and virulence noted above, we opted to explore whether such an association existed by investigating whether the EPS preparations from single and multi-motif differed in their effects on macrophages. Over the years, a significant body of literature has associated a detrimental impact of cryptococcal polysaccharide on macrophage function (40–45). However, the effects of structurally different polysaccharides have not been systematically examined. Given that cetyltrimethylammonium bromide (CTAB) precipitation, ultrafiltration, and freeze-drying processes (32, 46) can affect polysaccharide structure, we focused on comparing the activity of native EPS, where the media is only filtered through a 0.22 µm pore to avoid introducing structural changes. This process is essential due to the fact that immunological responses and EPS structure can be modified by the purification techniques (32, 47).

Host effector cell mitochondrial activity, nitrite, and ROS production (48, 49) was significantly altered positively (stimulated) or negatively (repressed) when incubated with EPS from different *C. neoformans* serotype A strains or EPS + β-glucans. DC cells had their nitrite production suppressed by the H99 or KN99α EPS when compared with control cells. M1 macrophages had their levels of ROS stimulated when the same EPS type strains were compared with the control cells. The β-glucans were chosen as a stimulus instead of heat-inactivated *cap59*Δ because the heating of *cap59*Δ cells could release intracellular proteins and cell wall proteins that could potentially interfere with the analysis. Moreover, β-glucans are more favorable than LPS, as LPS and GXM serve as apoptotic triggers for activated M1 macrophages (50). The extent of cell activation was also modulated by the EPS and EPS β-glucans, affecting mitochondrial activity, nitrite production, and reactive oxygen species (ROS) generation. To simulate the initial response of unactivated M0 macrophages, GM-CSF was employed as a stimulus from innate lymphoid cells (ILC) (51). Our findings indicate that structurally diverse EPS exert different effects on macrophages, which aligns with our previous observations that single and multi-motif cryptococcal strains exhibit differences in virulence.

Survival experiments using different routes of infection showed differences in virulence and pathogenicity for the four cryptococcal strains. Fungal load measurements revealed remarkable differences between the four strains evaluated and the inoculation route. For the IN-infected mice, the presence of a single-motif in the capsule was associated with reduced virulence for strains Mu-1 or 24064, as few or no CFUs were found in the lung or brains. In contrast, although higher CFU loads were present in the lungs and brains of mice infected with the multiple motif strains H99 or KN99α (16, 23, 52–56). For the IV-infected mice, a similar result was observed for a 5-day infection interval, which was chosen based on the survival data. Overall, the results suggest that the mice were better at controlling and preventing fungal dissemination of the single-motif strains in the lungs of the mice. Based on our *in vitro* studies demonstrating significant immune interactions within a 2 h timeframe, we hypothesized that the initial immune response occurs within the first 12 h post-infection. This idea is further supported by our preliminary results, indicating that mice infected via the intratracheal route with various *C. neoformans* strains, including H99, KN99α, Mu-1, and 24064, showed significantly lower CFU recovery for the Mu-1 and 24064 strains than for the multi-motif strains H99 and KN99α.

Differences in inflammatory response paralleled differences in virulence. Cytokine levels in the lungs of mice infected via the intranasal route revealed elevated concentrations of IL-4 and IL-10 in those infected with the H99 strain, whereas mice infected with the Mu-1 strain exhibited increased levels of IL-1β and IFN-γ. In contrast, for intravascular infection, both H99 and KN99α strains elicited the highest levels of IL-6 and IL-1β. Interestingly, H99 infection resulted in a slightly elevated level of IL-4, whereas Mu-1 infected mice exhibited the highest levels of IFN-γ among the strains tested (57–59). These cytokine levels are in accordance with the existing literature, as *C. neoformans* has been reported to modulate the immune response by stimulating or repressing some cytokines (34, 57–59). For the strains expressing more than one motif, we noted a higher presence of anti-inflammatory and regulatory cytokines (57–59). The difference in the cytokine levels between the IN- or IV-infected mice may be related to the difference in the infection interval, since we infected for 20 days for all IN infections, 20 days for Mu-1 and 24064 IV infections, and 5 days for H99 or KN99α IV infections.

Histological data also showed differences in the inflammatory response of the multi-motif strains and single-motif strains (40–42). Mice infected with the multi-motif strains had a better preservation of lung structure, parenchyma, and alveoli but had numerous cryptococcal cells in lung tissue. For the single-motif strains, there were strong inflammatory infiltrates with no apparent cryptococcal cells in the lung parenchyma and alveoli (57–59). The flow cytometry analysis of the lung of IN-infected mice after 20 days showed that the presence of activated M1 macrophages and CD4+ lymphocytes is necessary for the reduction of fungal load and control of disease progression. This may support that different motifs in the *Cryptococcus* capsule may be responsible for modulating how the immune system fights the infection (57–61).

The prevalence of single versus multiple motif strains among *C. neoformans* isolates was characterized by NMR among 59 clinical strains, by the prior work of Cherniak (29) and our laboratory (62). Among this set, only five isolates have been found to have a single-motif GXM (8%), one from serotype D (ATCC 24067) expressing the M1, two from serotype A (Mu-1 and 24064) expressing the M2 motif, one from serotype B (409) expressing the M3 motif, and one from serotype C (kt24066) expressing the M4 motif (29, 62). If one adds to this set another 11 strains (two from veterinary sources, two from laboratories, and seven environmental isolates) that have multi-motif GXM, the prevalence of single-motif strains among those characterized by NMR (5/70) is even lower, at 7%. Lower prevalence of single-motif strains among clinical isolates is consistent with lower virulence. A similar argument was made to explain the lower prevalence of serotype D relative to serotype A strains (63, 64) and mating type alpha among cryptococcal clinical isolates (65).

We have considered possible mechanisms to explain how differences in *C. neoformans* polysaccharide complexity may affect virulence. NMR and molecular dynamics analysis of a synthetic repeating M2 motif implied that it was a relatively stiff structure (66). Changes in the motif composition can be anticipated to affect the flexibility of the polysaccharide and its interaction with host cellular receptors. In this regard, GXM from *C. neoformans* serotypes A was reported to interact with Dectin-1 and Dectin-2 (67), whereas serotypes AD and *C. gattii* serotype B have been reported to interact with Dectin-3 (68). For *C. neoformans* and *C. gattii,* the immunomodulatory effects of GXM correlated with polysaccharide molecular diameter, implicating structure as a variable in immunological effects (47, 69). Since motif composition plays a crucial role in determining the structure of polysaccharides, and structurally different polysaccharides can interact with various immune receptors (14, 15), this differential interaction can result in varied activation of receptors, influencing the responsiveness of macrophages and dendritic cells, as well as the inflammatory response. Consequently, it is plausible to propose a mechanism through which the structural complexity of polysaccharides may correlate with differences in virulence.

In summary, two strains equipped with capsules composed of only M2 motif polysaccharides presented lower virulence and pathogenicity in comparison to multi-motif strains despite comparable expression of other virulence factors. Considering that capsular polysaccharides of *C. neoformans* can alter the host immune response, our data suggest that this interaction may be highly dependent on the motif composition of cryptococcal GXM. This study provides the first evidence that a *C. neoformans* polysaccharide capsule composed exclusively of the M2 motif of GXM is not sufficient to sustain infection or promote full virulence despite these strains having comparable expressions of the major virulence factors. Our analysis is limited by the fact that current technology prevents the generation of isogenic cryptococcal strains with variations solely in motif composition. Nevertheless, the findings align well with the observed low prevalence of single-motif strains among clinical isolates. Our results highlight the need for more in-depth studies using other single-motif strains combined with a more robust immunological analysis to understand the relationship between polysaccharide complexity and virulence in *C. neoformans*. However, such studies will depend on the future isolation and characterization of more single-motif strains from clinical isolates, which will require a significant new effort in studying polysaccharide structural composition. Despite the limitations inherent in this study, our results showing a repression in the nitrite production of DC cells by the EPS from the multi-motif H99 and KN99α strains, the stimulation in ROS production in M1 macrophages and DC cells by the EPS from the multi-motif H99 and KN99α strains, and the higher presence of CD4+ lymphocytes in the lung of mice infected with the single-motif strains Mu-1 and 24064 implicate capsular polysaccharide complexity as yet another variable that contributes to the cryptococcal virulence composite.

## MATERIALS AND METHODS

### *C. neoformans* strains and media growth conditions

*C. neoformans* strains multi-motif H99 and KN99α, single-motif (motif M2) Mu-1 and ATCC-24064 (24064), *cap59*∆ (acapsular strain) a *C. neoformans var. neoformans* variation of the wild-type isolate B-4131 (36), H99 *ure1*Δ and KN99α *ure1*Δ (urease deletion) and H99 *lac1*Δ and KN99α *lac1*Δ (laccase deletion), stored in −80°C in glycerol stock from our laboratory and were used in all experiments. *C. neoformans* from frozen stocks were cultivated in different media, as yeast extract peptone dextrose (YPD) (BD DIFCO, Franklin Lakes, NJ, USA), Minimal media (MM) (15 mM D-glucose, 10 mM MgSO_4_ × 7•H_2_O, 20.3 mM KH_2_PO_4_, 3 mM glycine, 10 mg/mL thiamine, pH 5.5) or Sabouraud dextrose broth (SAB) (BD DIFCO, Franklin Lakes, NJ, USA). Liquid cultures were maintained at 30°C in a spinning wheel at 150 rpm for 48 h and re-cultured for another 24 h at 30°C in a spinning wheel at 150 rpm. The solid agar cultures were grown at 30°C in Sabouraud dextrose agar (SDA) (BD DIFCO, Franklin Lakes, NJ, USA) complemented with 1% penicillin/streptomycin (Pen/Strep) (Gibco, Waltham, MA, USA) for 72 h.

### EPS production and isolation

*C. neoformans* H99 and KN99α (multi-motif) and single M2 motif Mu-1 and 24064 were cultivated in 50 mL of MM for 1 week at 30°C under agitation (150 rpm). The acapsular strain *cap59*∆ was used as a control and cultivated under the same conditions. After 1 week, the medium was filtered through a 0.22 µm Steriflip (Sigma-Aldrich, St. Louis, MO, USA), the cells were discarded, and the MM supernatant containing the EPS of each strain was kept at 4°C. The presence of protein in the EPS was verified by silver stain (Pierce, Thermo Fisher Scientific, Waltham, MA, USA) using a Mini-PROTEAN TGX Precast Gels (Bio-Rad, Hercules, CA, USA).

Endotoxin (LPS) levels were measured using the Chromogenic Endotoxin Quant Kit (Pierce, Thermo Fisher Scientific, Waltham, MA, USA), and no detectable amount of LPS was found.

### EPS 1D proton nuclear magnetic resonance (NMR)

1D [1H] NMR (29, 62) data were collected on a 600 MHz Bruker Avance II (Bruker, Billerica, MA, USA), equipped with a triple resonance, TCI cryogenic probe (Bruker, Billerica, MA, USA), and *z*-axis pulsed field gradients. Spectra were collected at 60°C, with 128 scans and a free induction decay size of 84,336 points. Standard Bruker pulse sequences were used to collect the 1D data (p3919gp and zggpw5). Data were processed in Topspin version 3.5 (Bruker, Billerica, MA, USA) by truncating the free induction decay to 8192 points using a squared cosine bell window function and zero filling to 65,536 points. All NMR samples contained DSS-d6 for chemical shift calibration and peak intensity comparisons. GXM motifs were identified based on peak chemical shifts in the structural reporter group (SRG) region (5.0-5.4 ppm). GXM motif percentages for multi-motif expressing strains were determined by comparison of motif-distinguishing peak integration compared with that of the entire SRG region.

### EPS size and polydispersity index (PDI) measurements

EPS size and PDI were analyzed by dynamic light scattering (DLS) in the Zeta Plus (Brookhaven Instruments Corporation, Nashua, NH, USA). The mean of five technical replicates for each EPS strain type was used to calculate the size and PDI.

### Phenol-sulfuric acid (PSA) assay for extracellular polysaccharides quantification

To compare the concentration of cryptococcal EPS, a modified phenol-sulfuric acid (PSA) assay was used (32, 70, 71). Samples were hydrolyzed with 4 M HCl at 100°C for 30  min. After cooling on ice, 80  µL of the hydrolyzed sample was mixed with 3.6  mL of the color reagent (0.5  mL of 5% [wt/vol] phenol + 2.5  mL concentrated sulfuric acid) in an ice-water bath. After thorough mixing, samples were incubated in a hot water bath for color development. Absorbance was measured at 490 nm with the microtiter plate reader SpectraMAX 340 Tunable Microplate Reader (Molecular Devices Ltd, San Jose, CA, USA). The mean of three technical replicates for each EPS strain type was used to calculate the EPS concentration.

### *C. neoformans* cell body and capsule size measurement

The cryptococcal cell body and capsule size measurements were performed using India ink negative staining, after growth in different media (YPD, MM, or SAB). Images were taken on an Olympus AX70 microscope (Olympus Optical Company, Center Valley, PA, USA) and then analyzed using the Quantitative Capsule Analysis program or manually using ImageJ (NIH, Bethesda, MD, USA). The capsule size was established as a capsule-to-cell body diameter ratio. The mean of fifty cells for each strain was used to calculate the body and capsule size.

### Biofilm assay

*C. neoformans* biofilm formation was evaluated using the crystal violet assay (Sigma-Aldrich, St. Louis, MO, USA) (72); 1 × 10^7^ yeast from different serotype A strains of *C. neoformans* (*cap59*∆, H99, KN99α, Mu-1, or 24064) were grown for 24 h at 30°C and 37°C in SAB on a 96-well plate. After 24 h, the wells were washed twice with 200 µL of 1× phosphate buffer saline (PBS) and then air-dried for 45 min. After the drying process, a volume of 110 µL of 0.4% crystal violet was added to the wells, and the plate was incubated at room temperature for 45 min. After the incubation, the plate was washed four times with 350 µL of sterile distilled water. After the washing, a volume of 200 µL of 95% ethanol was added to the wells, and the plate was incubated at room temperature for 45 min. After the incubation, a volume of 100 µL of ethanol plus crystal violet solution from each of the wells was transferred to a new 96-well plate and the amount of the crystal violet stain in the ethanol was assessed by measuring the absorbance of the solution with the microtiter plate reader SpectraMAX 340 Tunable Microplate Reader (Molecular Devices Ltd, San Jose, CA, USA) at 595 nm. The mean of eight biological replicates for each strain was used to calculate the biofilm production.

### Hydrophobicity

Cell surface hydrophobicity (CSH) of five strains of *C. neoformans* (H99, KN99α, Mu-1, 24064, and *cap59*∆) was measured by the microbial adhesion to hydrocarbons (MATH) assay. The *C. neoformans* strains were grown in SAB for 24 h at 30°C in a spinning wheel at 150 rpm. After growth, the *C. neoformans* strains were washed twice with PBS at 3,000 rpm for 5 min. The *C. neoformans* strains were resuspended in 3 mL of PBS at an estimated optical density (OD 600) of 0.2*–*0.4, vortexed with 400 µL hexadecane (Sigma-Aldrich) for 60 s, and left to sit at room temperature for 2 min, to allow the layers to separate. After the rise of the hydrophobic layer, the bottom layer (aqueous layer) was transferred to a 96-well plate, and the OD 600 was measured in the SpectraMAX 340 Tunable Microplate Reader (Molecular Devices Ltd). Due to their hydrophobicity, a proportion of cells enter the hydrophobic layer, resulting in a decreased absorbance in the aqueous layer.

CSH was calculated as


$$CSH\%=\frac{\left(A0-A1\right)}{A0}\;\times \;100$$

where A0 = absorbance of cells before the addition of the hexadecane and A1 = absorbance of the aqueous layer after cells entered the hydrophobic layer. The mean of three biological replicates for each strain was used to calculate the hydrophobicity of each strain.

### Growth curve

Growth patterns were evaluated by incubating 1 × 10^7^ *C. neoformans* yeast cells of each strain in a 96 wells microplate with YPD, SAB, or MM liquid media at 30°C or 37°C, under agitation, and the absorbance was measured in a microtiter plate reader SpectraMAX 340 Tunable Microplate Reader (Molecular Devices Ltd, San Jose, CA, USA) at 600 nm for 72 h. The mean of four biological replicates for each strain was used to calculate the growth curve; sterile YPD, SAB, or MM was used as a blank.

### Laccase measurement

Laccase measurement was evaluated by incubating 1 × 10^7^ *C. neoformans* yeast cells of each strain in MM supplemented with 1 mM of 2,2′-azinobis (3-ethylbenzothiazoline-6-sulfonic acid)-diammonium salt (ABTS) (Sigma-Aldrich, St. Louis, MO, USA). The absorbance was measured in a microtiter plate reader SpectraMAX 340 Tunable Microplate Reader (Molecular Devices Ltd, San Jose, CA, USA) at 734 nm for 72 h. The mean of six biological replicates for each strain was used to calculate the laccase curve; sterile MM was used as a blank, and H99 *lac1*Δ and KN99α *lac1*Δ were used as negative controls.

### Urease measurement

Urease measurement was evaluated by cultivating each strain in urea broth (UB) (2) composed of 1 g of peptone, 5 g of NaCl, 2 g of KH_2_PO_4_, 12 mg of phenol red, 1 g of glucose, 20 g of urea, and 1 L of distilled water. Each strain was incubated in 15 mL tubes; 1 × 10^7^ *C. neoformans* yeast cells were added to 5 mL of UB in triplicate, for 72 h in a spinning well at 30°C. After 24, 48, and 72 h, the tubes were centrifuged at 3,000 rpm for 5 min, and 300 µL of each tube was removed and placed in a 96-well microplate (100 µL per tube), and 300 µL of new UB media was added to the tubes. The absorbance was measured in a microtiter plate reader SpectraMAX 340 Tunable Microplate Reader (Molecular Devices Ltd, San Jose, CA, USA) at 560 nm. The mean of nine biological replicates for each strain was used to calculate the urease production, sterile UB was used as a blank, and H99 *ure1*Δ and KN99α *ure1*Δ were used as a negative control.

### Phospholipase activity

The phospholipase presence and activity were measured by plating 1 × 10^7^ *C. neoformans* yeast cells in 100 µL in SDA supplemented with 10% egg yolk (HiMedia, Kennett Square, PA, USA).

The precipitation index was calculated by the following formula:


$$Precipitation\;index=\frac{HD}{CD}$$

where HD = halo diameter and CD = colony diameter.

The mean of three biological replicates was used to calculate the final precipitation index.

### Melanin production

For melanin production (73) determination, 1 × 10^7^ *C. neoformans* yeast cells were streaked in MM agar plates supplemented with 1 mM L-DOPA (Sigma-Aldrich, St. Louis, MO, USA) or in six-well cell culture plates with 2 mL per well of MM liquid culture supplemented with 1 mM L-DOPA (Sigma-Aldrich, St. Louis, MO, USA) and incubated steady or under agitation respectively for 72 h at 30°C. The plates were then scanned (CanoScan 9000F scanner; Melville, NY, USA) at 600 dpi, and the mean gray value was measured using Image J (NIH, Bethesda, MD, USA).

### Re-encapsulation of acapsular *C. neoformans* with extracellular polysaccharides

The re-encapsulation (21, 34) was performed by incubating the *cap59*∆ strains with the EPS from the H99, KN99α, Mu-1, or 24064 at room temperature (24°C) for 1 h under rotation. For all experiments using re-encapsulated yeast, the groups were divided into WT (wild type or original cells) H99, KN99α, Mu-1, 24064, and *cap59*∆ or EPS (*cap59*∆ cells re-encapsulated with EPS from the WT cultures in MM).

### Immunofluorescence

Re-encapsulation was confirmed by immunofluorescence; after incubation with the EPS, the cells were centrifuged and washed twice with PBS to remove any trace of free EPS. The cells were then blocked with 2% bovine serum albumin (BSA) (MP biomedics, Santa Ana, CA, USA) in PBS at room temperature for 1 h under rotation. After the blocking, the cells were incubated with 0.02 µg/mL of 18B7 mAb. The cells were then centrifuged and washed with PBS; the cells were then incubated with a secondary IgG (H + L) conjugated with FITC 1030-02 (Southernbiotech, Birmingham, AL, USA) at a 1:100 dilution and 2 µg/mL of Uvitex 2B (Polysciences, Inc., Warrington, PA, USA) at room temperature for 1 h under rotation. The cells were then centrifuged, washed, centrifuged again, and resuspended in one drop of ProLong (Thermo Fisher Scientific, Waltham, MA, USA), and the cells were visualized in the Leica Thunder microscope system (Leica, Deerfield, IL, USA).

### Phagocytosis assays

For the phagocytosis experiments, J774 ATCC macrophage-like cells were utilized; the cells were maintained in Dulbecco’s modified Eagle’s medium (DMEM) (Corning, Corning, NY, USA) and 10% heat-inactivated fetal bovine serum (FBS) (Cytiva, Marlborough, MA, USA). Approximately 24 h prior to the phagocytosis experiments, 2.5 × 10^5^ J774 cells were plated in 24-well plates; the phagocytosis was performed with 15 × 10^5^ *C. neoformans* cells in the following groups: WT (*cap59*∆, H99, KN99α, Mu-1, and 24064) and EPS (*cap59*∆, H99, KN99α, Mu-1, and 24064). The final effector-to-target (E:T) ratio was 1:3 (J774 cells have a 24 h doubling time). After 2 h of interaction, the wells were washed twice with 1 mL of PBS per well, and the macrophages and fungi were fixed with ice-cold methanol for 30 min at 4°C. The wells were then washed twice with 1 mL of PBS per well, and the plates were kept in the dark at 4°C. The cells were then blocked with 2% bovine serum albumin (BSA) (MP biomedics,Santa Ana, CA, USA) in PBS at room temperature for 1 h under rotation. After blocking, the cells were incubated with 18B7 antibody at a 1:100 dilution. The cells were then centrifuged and washed with PBS. The cells were then incubated with a secondary antibody anti IgG (H + L) conjugated with FITC 1030-02 (Southernbiotech, Birmingham, AL, USA) at 1:100 dilution, and 2 µg/mL of Uvitex 2B (Polysciences, Inc., Warrington, PA, USA) was used at room temperature for 1 h under rotation. The cells were washed, and one drop of ProLong (Thermo Fisher Scientific, Waltham, MA, USA) was added, and the cells were visualized in the Leica Thunder microscope system (Leica, Deerfield, IL, USA). To evaluate the phagocytosis, the total Field Of View (FOV) fluorescent area inside a range of approximately 0.9 mm^2^ was calculated. Non-phagocytosed yeast was removed by the washing steps before fixation.

### Mice

A/J or C57BL/6J female mice 6 to 8 weeks old (74–77) were acquired from the Jackson laboratory (Bar Harbor, ME, USA) and maintained at the Johns Hopkins University Bloomberg School of Public Health (JHSPH) animal facility with access to food and water *ad libitum*.

### Mitochondrial activity

Mitochondrial activity was measured using 5 × 10^5^ J774. ATCC macrophage-like cells were incubated with EPS from different Serotype A strains of *C. neoformans* (*cap59*∆*,* H99, KN99α, Mu-1, or 24064) or EPS + 10 µg/mL of β-glucans (Zymosan A [Sigma-Aldrich, St. Louis, MO, USA]). Mitochondrial activity was measured by the formation of formazan after 2 h post-addition and incubation of 3-(4,5-dimethylthiazol-2-yl)-2,5-diphenyltetrazolium bromide (MTT; Sigma-Aldrich, St. Louis, MO, USA) to each well following the manufacturer’s instructions. Control cells (c) were incubated with cell culture media only or cell culture media + β-glucans (10 µg/mL).

### Bone marrow-derived macrophages (BMDM) and bone marrow-derived dendritic cells (DCs)

BMDMs and DCs (78, 79) were generated by differentiation of bone marrow myeloid cells into macrophages or dendritic cells *in vitro* using the following protocol. C57BL/6J female mice were euthanized by CO_2_ asphyxiation, the femurs and tibias were excised aseptically and kept in ice-cold harvesting media composed of Dulbecco’s modified Eagle medium (DMEM) (Corning, Corning, NY, USA) and 10% heat-inactivated fetal bovine sera (FBS) (Cytiva, Marlborough, MA, USA) until all four bones were excised. The tips of each bone were cut, and the cells were collected in a 50 mL conical tube by flushing the bone marrow out of the bones using 10 mL of ice-cold harvesting media and a 26 G needle. The cells were then centrifuged at 3,000 rpm for 10 minutes at 4°C. After tAhe centrifugation, the cells were suspended in differentiation media, composed of FBS 10%, L929 supernatant 20% ATCC, GlutaMAX 1% (Gibco, Waltham, MA, USA), MEM Non-essential Amino Acids 1% (Corning), penicillin/streptomycin (Pen/Strep) 1% (Gibco, Waltham, MA, USA), HEPES 1% (Corning), β-mercaptoethanol 0.1% (Gibco), and DMEM (Corning, Corning, NY, USA) for the BMDM cells, and for the DC cells the differentiation media were composed of FBS 10% (Cytiva, Marlborough, MA, USA), 1% (vol/vol) penicillin/streptomycin (Gibco, Waltham, MA, USA), 30 ng/mL of GM-CSF, and 15 ng/mL of IL-4 (Invitrogen, Waltham, MA, USA) and RPMI-1640 (Corning, Corning, NY, USA). The cells were then seeded in a 100 mm Non-TC-treated Culture Dish (Corning) and were maintained in an incubator at 37°C, 5% CO_2_, and 70% relative humidity for one week. The media was changed every 3–4 days. The cells were later divided into the following groups: BMDM = M0 macrophages (no activation or differentiation), GM-CSF = M0 macrophages stimulated with 20 ng/ml of GM-CSF overnight, M1 = M1 macrophages activated and differentiated overnight with 100 U/ml of IFN-γ and 500 ng/ml, and DCs = dendritic cells.

### Nitrite and reactive oxygen species (ROS) production

To evaluate the effect of the different strains, EPS in the production of nitrite and ROS, 5 × 10^5^ macrophage cells or 5 × 10^5^ dendritic cells were incubated for 12 h at 37°C and 5% CO_2_. The cells were incubated with a mix of 1:1 of EPS from different serotype A strains of *C. neoformans* (*cap59*∆, H99, KN99α, Mu-1, or 24064) or EPS + 10 µg/mL of β-glucans (Zymosan A [Sigma-Aldrich, St. Louis, MO, USA]), and a 2× concentrated phenol red-free cell culture medium (DMEM; Sigma-Aldrich, St. Louis, MO, USA) for the macrophages and RPMI-1640 (Cytiva, Marlborough, MA, USA) for the dendritic cells. Control cells (c) were incubated with only cell culture media (DMEM or RPMI-1640) or cell culture media (DMEM or RPMI-1640) + β-glucans (10 µg/mL). Nitrite production was evaluated with the Griess Reagent Kit (Invitrogen, Waltham, MA, USA) following the manufacturer’s instructions. The ROS production was evaluated with Amplex Red Hydrogen Peroxide/Peroxidase Assay Kit (Invitrogen, Waltham, MA, USA) following the manufacturer’s instructions. The following groups were utilized: BMDM = M0 macrophages (no activation or differentiation), GM-CSF = M0 macrophages stimulated with 20 ng/mL of GM-CSF overnight, M1 = M1 macrophages activated and differentiated overnight with 100 U/mL of IFN-γ and 500 ng/mL, and DCs = dendritic cells. A total of 5 × 10^5^ were plated per cell type in four biological replicates. Control cells (c) were incubated with cell culture media only or cell culture media + β-glucans (10 µg/mL).

### Murine infection

Before the infection, *C. neoformans* cells from liquid cultures were washed twice with PBS, and an aliquot of 10 µL was placed in a hemocytometer with trypan blue (Sigma-Aldrich, St. Louis, MO, USA) to count the number of yeast cells. For the intranasal infection (IN), mice were put under anesthesia using 100 mg/kg of ketamine (Covetrus, OH, US), 10 mg/kg of Xylazine (Covetrus, OH, USA), and 20 µL of PBS containing 5 × 10^5^ cryptococcal cells (10 µL per nostril) were inoculated. For the intravenous infection (IV), 100 µL of PBS containing 5 × 10^5^ cryptococcal cells was inoculated in the lateral tail vein of the mice. Mice were infected with strains Mu-1 and 24064 15 days prior to infection with strains H99 and KN99α. The euthanasia and cytokine quantification were done at the same time. For the IV and IN infections, five mice per group were randomly organized into the following groups: Sham (not infected) and the infected groups with H99, KN99α, Mu-1, and 24064. Separate experiments were conducted for survival and fungal pathogenesis. Results represent one of two independent replicates with similar results and trends among them. For intratracheal infection (IT), mice were put under anesthesia using 100 mg/kg of ketamine (Covetrus, OH, US) and 10 mg/kg of Xylazine (Covetrus, OH, US); 50 µL of PBS containing 5 × 10^5^ cryptococcal cells was inoculated via tracheotomy directly into the mouse trachea. Five mice per group were randomly organized into the following groups: H99, KN99α, Mu-1, and 24064. Mice were euthanized after 12 h of infection. The results represent one replicate.

### Tissue fungal burden

Euthanasia time was decided based on the survival curve. We aimed to get the infection at its peak, but with enough time prior to any illness manifestation of the animal and their death.

After euthanasia, the lungs and brains were aseptically removed, transversal sections of the lung tissue were randomly collected for histological processing, and the remaining tissue was weighed. The lungs and brains were homogenized in 2 mL of PBS, and 100 µL was plated in SDA media supplemented with 1% Pen/Strep. After 72 h of incubation at 30°C, CFUs were counted.

### Cytokine levels

For cytokine quantification, lung homogenates were aliquoted into microtubes with 500 µL of Roche cOmplete protein inhibitor (Roche, Indianapolis, IN, USA). Cytokine analysis was performed by enzyme-linked immunosorbent assay (ELISA) using a commercial kit (Thermo Fisher Scientific, Waltham, MA, USA) for the following cytokines: IL1-β, IFN-γ, IL-4, IL-6, and IL-10, following the manufacturer’s instructions.

### Lung histology

To evaluate the lung tissue after infection, lung transversal sections were fixed in 10% buffered formalin and embedded in paraffin using standard protocol. Histological slides were stained by the Johns Hopkins University Reference Histology Laboratory with hematoxylin and eosin (HE) for evaluation of the cellular structure of the organ, silver staining was performed to evaluate the presence of the fungal cell wall, and Masson staining was performed to evaluate collagen deposition. Slides were scanned by the Johns Hopkins University Oncology Tissue and Imaging Service (OTIS) Core Laboratory (Grant number, P30 CA006973) using the NanoZoomer S360MD Slide scanner (Hamamatsu Corporation, Bridgewater, NJ, USA). All original scanned slides are available upon request.

### Flow cytometry

To identify the immune cell population in the mice’s lungs after IN infection, a flow cytometry was performed, and the data acquisition was achieved in the Bloomberg Flow Cytometry and Immunology Core using the BD FACSymphony A3 (Becton, Dickinson and Company, Franklin Lakes, NJ, USA). Data analyses were performed in FlowJo 10 software (Becton, Dickinson, and Company, Franklin Lakes, NJ, USA). After euthanasia, the whole right lung was aseptically excised, minced, and digested for 1 h at 37°C under agitation in RPMI-1640 (Gibco, Waltham, MA, USA) supplemented with Collagenase D 150 U/mL (Roche, Indianapolis, IN, USA), DNase I Solution 50 U/mL (Roche, Indianapolis, IN, USA), and 1% Pen/Strep. After digestion, cells were filtered through a 40 µm cell strainer, and the red blood cells were lysed with eBioscience 1× RBC Lysis Buffer (Invitrogen, Waltham, MA, USA). Antibody staining was performed as indicated by the antibody (Table S1) manufacture company. Briefly after the addition of the antibodies, the cells were incubated for 30 min at 4°C in the dark. Cells were then washed in FACS buffer (Becton, Dickinson and Company, Franklin Lakes, NJ, USA) and fixed with ice-cold 4% paraformaldehyde for 10 min at room temperature. The gating strategy is shown in Fig. S8.

### Statistical analysis

Analysis of variance (ANOVA) for multiple comparisons (all groups against all groups or all groups against the control) or student *t*-test (pairwise comparison) was performed, followed by Tukey or Dunnett post-tests, respectively, using Graph Pad Prism 10. Multiple comparisons were corrected by Šídák’s multiple comparisons test. *P*-values were considered significant when *P* ≤ 0.05, and error bars were used representing the standard error of the mean (SEM).

## SUPPLEMENTAL MATERIAL

Supplemental figures and tables mbio.02646-25-s0001.docx Fig. S1 to S8; Tables S1 to S6.

ASM does not own the copyrights to Supplemental Material that may be linked to, or accessed through, an article. The authors have granted ASM a non-exclusive, world-wide license to publish the Supplemental Material files. Please contact the corresponding author directly for reuse.
