# Supplementary material for: Cryptococcus neoformans serotype A virulence and pathogenicity are capsular glucuronoxylomannan (GXM) motif composition dependent
Source: mBio. 2025 Sep 29;16(11):e02646-25. doi: 10.1128/mbio.02646-25 (PMC12607832; doi:10.1128/mbio.02646-25)
Supplement: Supplemental figures and tables — Fig. S1 to S8; Tables S1 to S6. [file mbio.02646-25-s0001.docx]

Table S1. Antibodies, colors and manufacturing companies.

| Cat N° | Color | Antibody | Clone | Company |
| --- | --- | --- | --- | --- |
| 100217 | PerCP/Cyanine5.5 | anti-mouse CD3 | 17A2 | Bio Legend |
| 100429 | Alexa Fluor® 700 | anti-mouse CD4 | GK1.5 | Bio Legend |
| 612898 | BUV805 | anti-mouse CD8a | 53-6.7 | BD |
| 104731 | Brilliant Violet 650™ | anti-mouse CD80 | 16-10A1 | Bio Legend |
| 101291 | Spark UV™ 387 | anti-mouse CD11b | M1/70 | Bio Legend |
| 137027 | Pacific Blue™ | anti-mouse CD68 | FA-11 | Bio Legend |
| 121511 | Alexa Fluor® 488 | anti-mouse CD83 | Michel-19 | Bio Legend |
| 105013 | PE/Cyanine7 | anti-mouse CD86 | GL-1 | Bio Legend |
| 156709 | PE/Dazzle™ 594 | anti-mouse CD163 | S15049F | Bio Legend |
| 141729 | Brilliant Violet 785™ | anti-mouse CD206 | C068C2 | Bio Legend |
| 117310 | APC | anti-mouse CD11c | N418 | Bio Legend |
| 111704 | PE | anti-mouse F4/80 | W20065D | Bio Legend |
| 108433 | Brilliant Violet 421™ | anti-mouse Ly-6G/Ly-6C(GR-1) | RB6-8C5 | Bio Legend |
| 107639 | Brilliant Violet 605™ | anti-mouse I-A/I-E (MHCII) | M5/114.15.2 | Bio Legend |
| 423105 | Zombie NIR™ Fixable Viability Kit | |  | Bio Legend |

Table S2. Chemical shift of peaks belonging to GXM motifs

| Assign | H99 | KN99a | Mu-1 | 24064 |
| --- | --- | --- | --- | --- |
| M4-Mc | 5.110 | 5.107 |  |  |
| M3-Mb | 5.150 |  |  |  |
| M2-Mc | 5.162 |  | 5.152 | 5.153 |
| M4-Ma/  M3-Ma/  M1-Mb/c | 5.222 | 5.195 |  | 5.217/ 5.223  doublet |
| M1-Ma |  | 5.232 |  |  |
| M2-Ma | 5.239 | 5.238 | 5.239 | 5.249 |
| M4-Mb/ M3-Ma | 5.329 | 5.327 |  |  |
| M2-Mb | 5.349 | 5.334 | 5.334 | 5.324 |


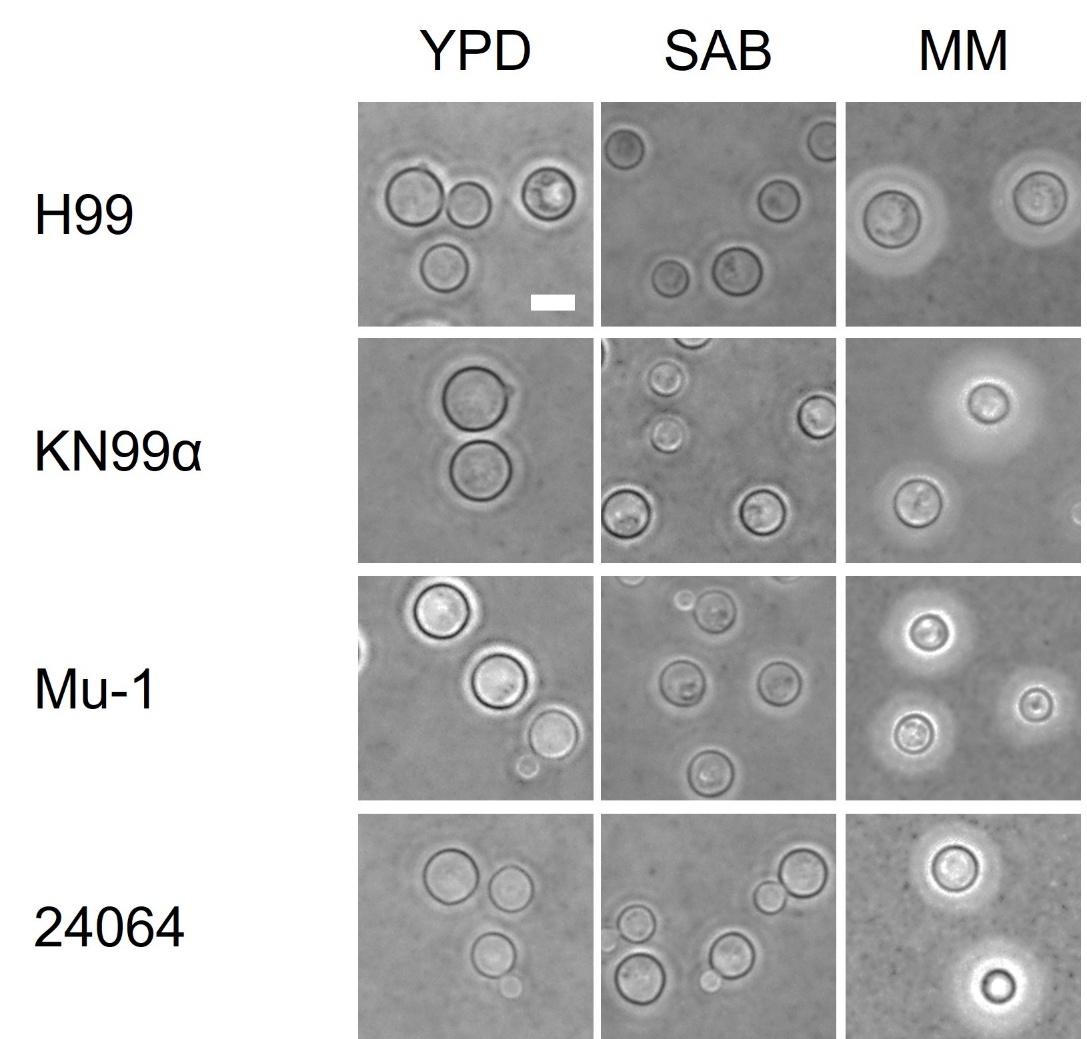


Fig. S1. India ink images of different Serotype A strains of C. neoformans (H99, KN99α, Mu-1, or 24064), cultivated in different culture media (YPF, SAB, and MM), showing body and capsule size. Whaite bar = 10 µm. 60× magnification.


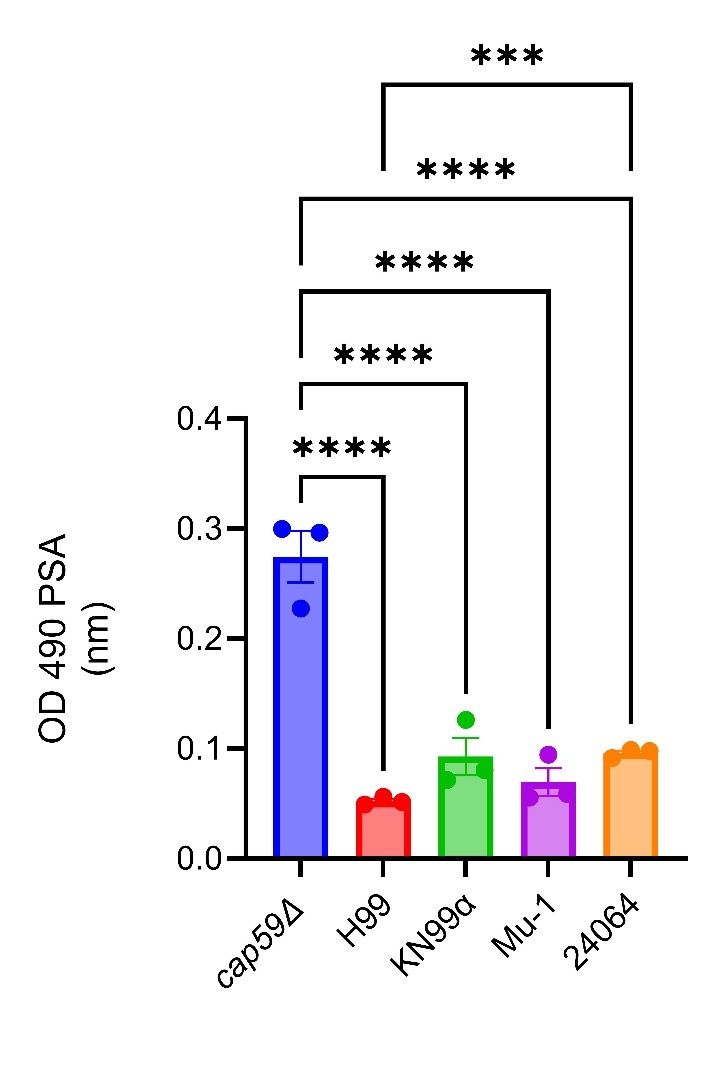


Fig. S2. Polysaccharide content quantification. Different Serotype A strains of C. neoformans (cap59∆, H99, KN99α, Mu-1, or 24064) were cultivated in MM for one week, media was then filtered, and polysaccharide content was quantified by the phenol-sulfuric acid method. Statistical analysis showed that all the different strains had similar levels of EPS n = 3 per group. *** = p<0.001, **** = p<0.0001 (t-test and or ANOVA). Multiple compressions were corrected by the Šídák's multiple comparisons test.


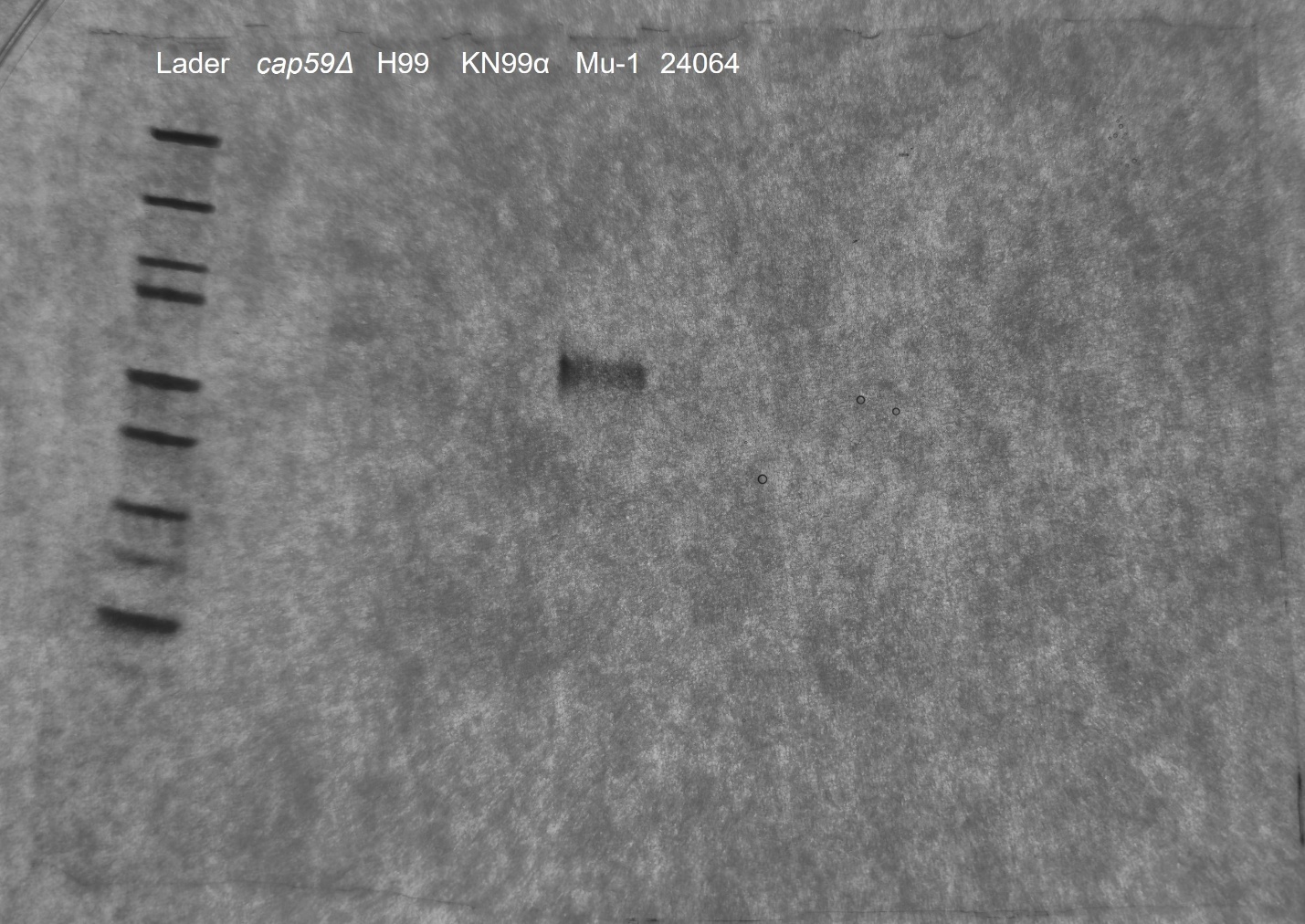


Fig. S3. Tris-Glycine (TGX) electrophoresis gel showing for proteins in the EPS polysaccharide from different Serotype A strains of C. neoformans (cap59∆, H99, KN99α, Mu-1, or 24064). A band is apparent in the Mu-1 that is absent in the others and this protein was not identified. Cells were cultivated in MM for one week.


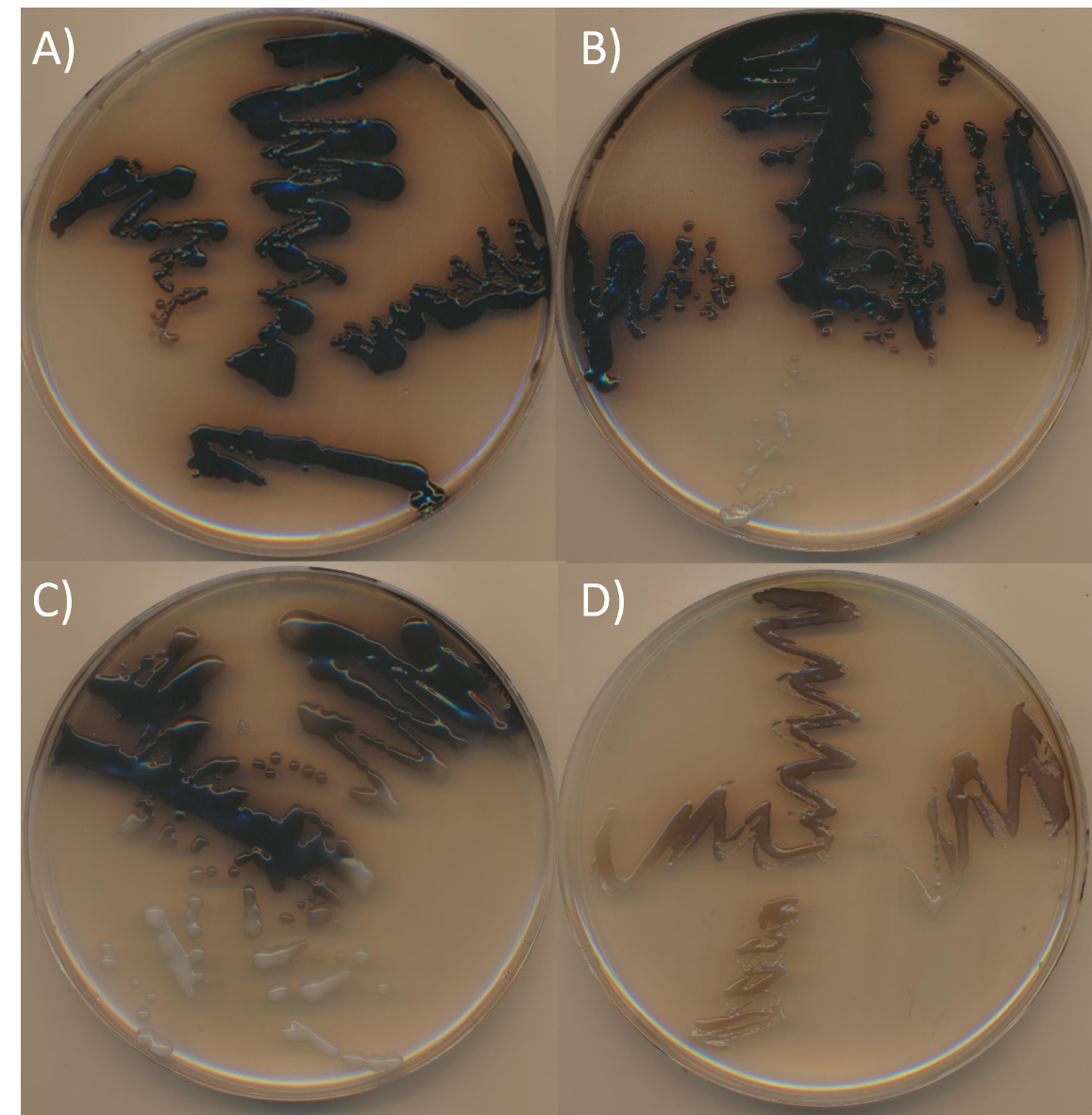


Fig. S4. L–DOPA plates showing melanin production of different Serotype A strains of C. neoformans. A) H99. B) KN99α. C) Mu-1. D) 24064.

**
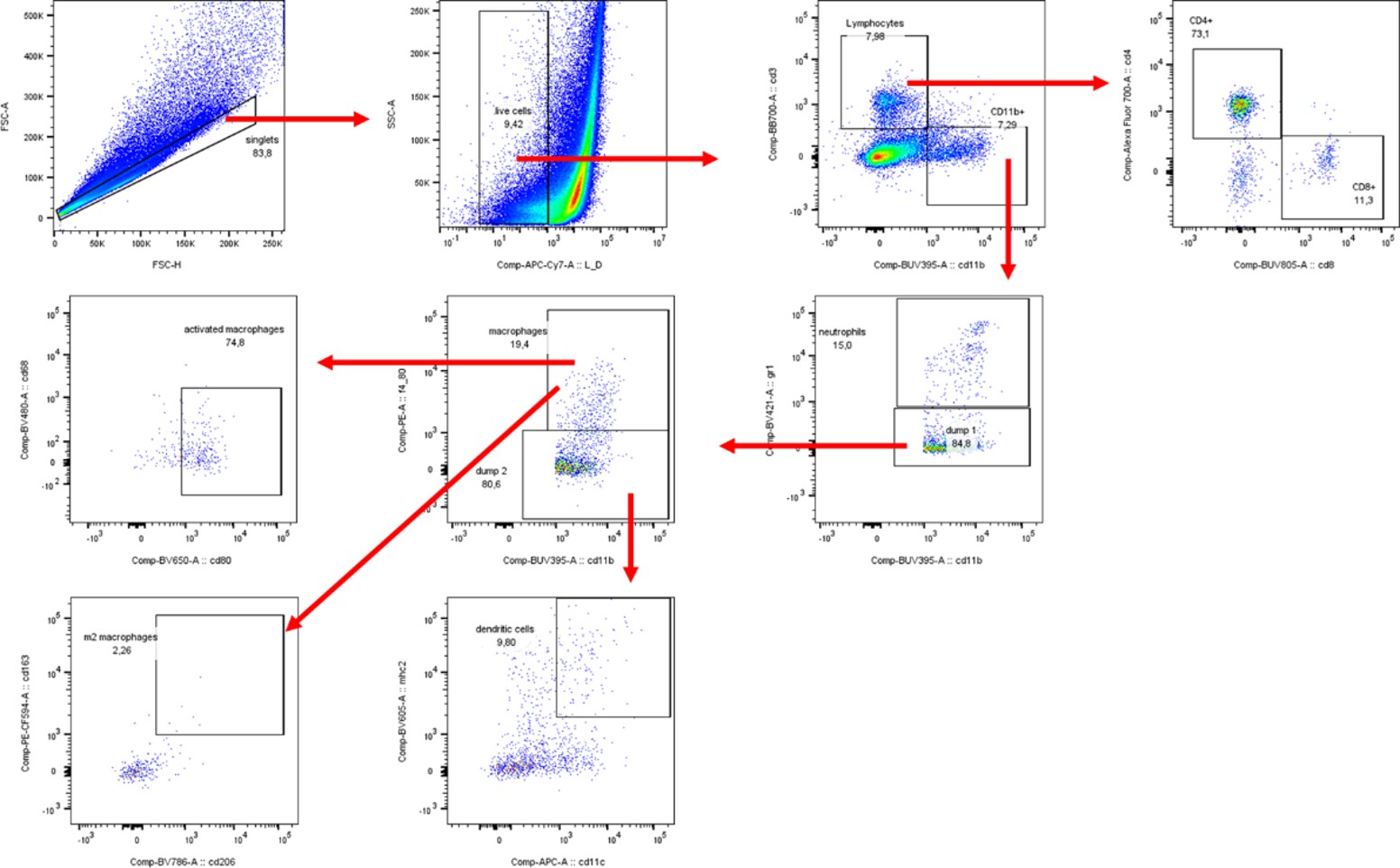
**

Fig. S5. Flow cytometry gating strategies.


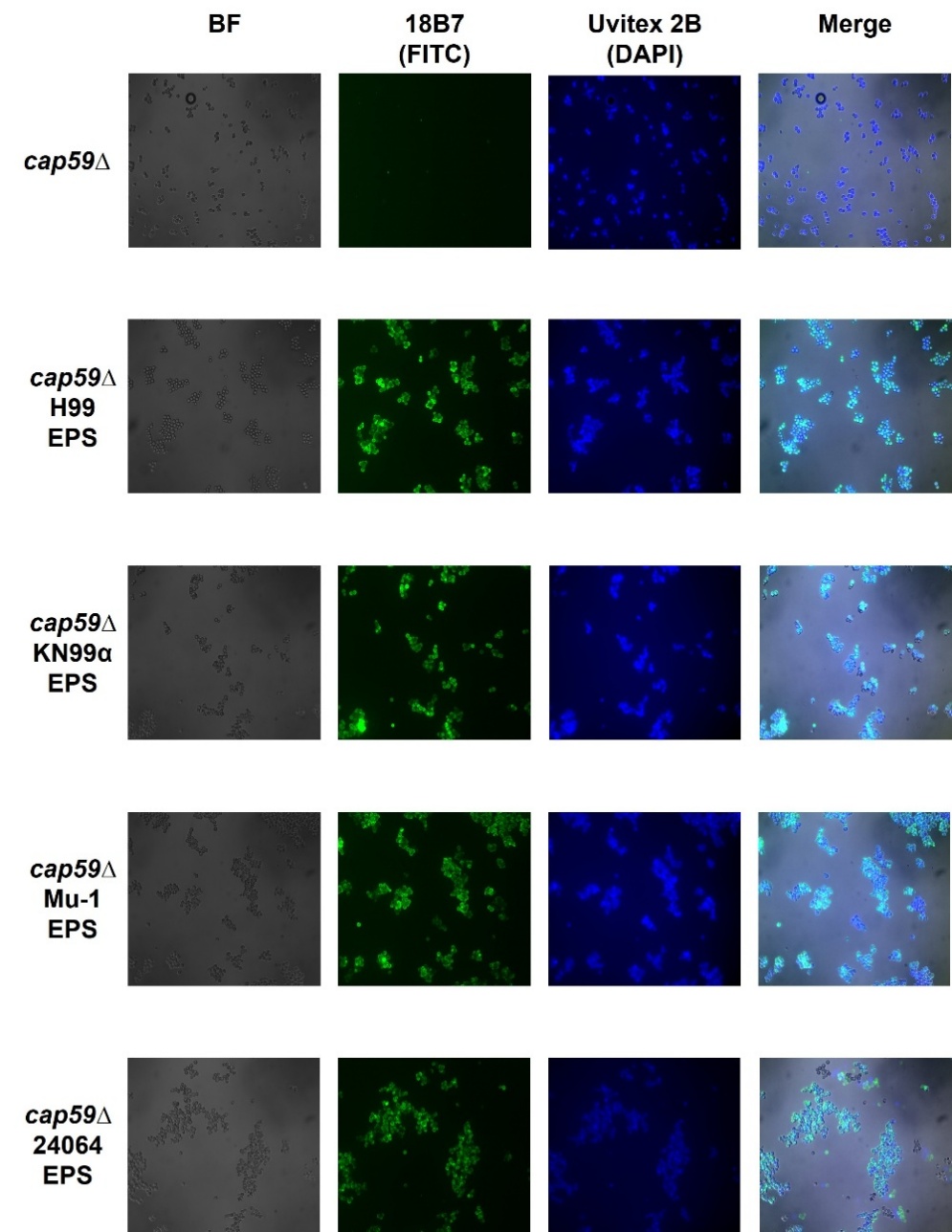


Fig. S6. Re-encapsulation immunofluorescence. Immunofluorescence images showing the ability of and acapsular strain (cap59∆) to create a “capsule” using exogenous EPS from different Serotype A strains of C. neoformans (cap59∆, H99, KN99α, Mu-1, or 24064). The monoclonal antibody 18B7 was used to stain the EPS and Uvitex 2B was used to stain the fungal cell wall. 40× magnification.


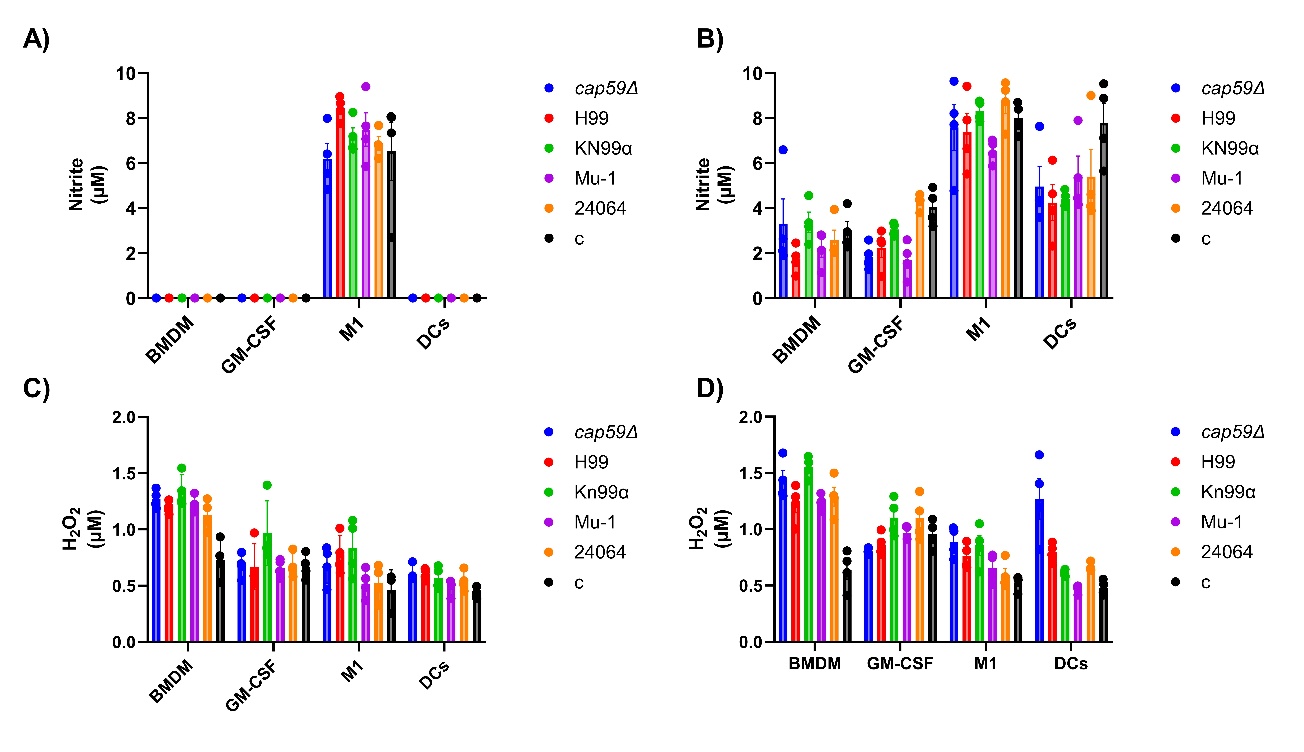


Fig. S7. Grouped comparison of nitrite or ROS production of different Serotype A strains of C. neoformans (cap59∆, H99, KN99α, Mu-1, or 24064) or EPS + β-Glucans (Zymosan A) and different cell types, activation status and stimulus. BMDM = M0 macrophages (no activation or differentiation) GM-CSF = M0 macrophages stimulated with 20 ng/mL of GM-CSF overnight, M1 = M1 macrophages activated and differentiated overnight with 100 U/mL of IFN-γ and 500 ng/mL. C = control cells (incubated with only cell culture media or cell culture media + β-Glucan).

Table S3. Nitrite EPS Statistical analysis

| Tukey's multiple  comparisons  test | Mean  Difference. | 95.00% CI of difference. | Below threshold? | Summary | Adjusted  P Value |
| --- | --- | --- | --- | --- | --- |
|  |  |  |  |  |  |
| *cap59Δ* |  |  |  |  |  |
| BMDM vs. GM-CSF | 0 | -0.9531 to 0.9531 | No | ns | >0.9999 |
| BMDM vs. M1 | -6.189 | -7.142 to -5.236 | Yes | **** | <0.0001 |
| BMDM vs. DCs | 0 | -0.9531 to 0.9531 | No | ns | >0.9999 |
| GM-CSF vs. M1 | -6.189 | -7.142 to -5.236 | Yes | **** | <0.0001 |
| GM-CSF vs. DCs | 0 | -0.9531 to 0.9531 | No | ns | >0.9999 |
| M1 vs. DCs | 6.189 | 5.236 to 7.142 | Yes | **** | <0.0001 |
|  |  |  |  |  |  |
| H99 |  |  |  |  |  |
| BMDM vs. GM-CSF | 0 | -0.9531 to 0.9531 | No | ns | >0.9999 |
| BMDM vs. M1 | -8.429 | -9.382 to -7.476 | Yes | **** | <0.0001 |
| BMDM vs. DCs | 0 | -0.9531 to 0.9531 | No | ns | >0.9999 |
| GM-CSF vs. M1 | -8.429 | -9.382 to -7.476 | Yes | **** | <0.0001 |
| GM-CSF vs. DCs | 0 | -0.9531 to 0.9531 | No | ns | >0.9999 |
| M1 vs. DCs | 8.429 | 7.476 to 9.382 | Yes | **** | <0.0001 |
|  |  |  |  |  |  |
| KN99α |  |  |  |  |  |
| BMDM vs. GM-CSF | 0 | -0.9531 to 0.9531 | No | ns | >0.9999 |
| BMDM vs. M1 | -7.186 | -8.139 to -6.233 | Yes | **** | <0.0001 |
| BMDM vs. DCs | 0 | -0.9531 to 0.9531 | No | ns | >0.9999 |
| GM-CSF vs. M1 | -7.186 | -8.139 to -6.233 | Yes | **** | <0.0001 |
| GM-CSF vs. DCs | 0 | -0.9531 to 0.9531 | No | ns | >0.9999 |
| M1 vs. DCs | 7.186 | 6.233 to 8.139 | Yes | **** | <0.0001 |
|  |  |  |  |  |  |
| Mu-1 |  |  |  |  |  |
| BMDM vs. GM-CSF | 0 | -0.9531 to 0.9531 | No | ns | >0.9999 |
| BMDM vs. M1 | -7.491 | -8.444 to -6.538 | Yes | **** | <0.0001 |
| BMDM vs. DCs | 0 | -0.9531 to 0.9531 | No | ns | >0.9999 |
| GM-CSF vs. M1 | -7.491 | -8.444 to -6.538 | Yes | **** | <0.0001 |
| GM-CSF vs. DCs | 0 | -0.9531 to 0.9531 | No | ns | >0.9999 |
| M1 vs. DCs | 7.491 | 6.538 to 8.444 | Yes | **** | <0.0001 |
|  |  |  |  |  |  |
| 24064 |  |  |  |  |  |
| BMDM vs. GM-CSF | 0 | -0.9531 to 0.9531 | No | ns | >0.9999 |
| BMDM vs. M1 | -6.869 | -7.822 to -5.916 | Yes | **** | <0.0001 |
| BMDM vs. DCs | 0 | -0.9531 to 0.9531 | No | ns | >0.9999 |
| GM-CSF vs. M1 | -6.869 | -7.822 to -5.916 | Yes | **** | <0.0001 |
| GM-CSF vs. DCs | 0 | -0.9531 to 0.9531 | No | ns | >0.9999 |
| M1 vs. DCs | 6.869 | 5.916 to 7.822 | Yes | **** | <0.0001 |

Table S4. Nitrite EPS+β-Glucan Statistical analysis

| Tukey's multiple  comparisons  test | Mean  Difference. | 95.00% CI   of difference. | Below threshold? | Summary | Adjusted  P Value |
| --- | --- | --- | --- | --- | --- |
|  |  |  |  |  |  |
| *cap59Δ* |  |  |  |  |  |
| BMDM vs. GM-CSF | 1.457 | -0.9300 to 3.843 | No | ns | 0.3793 |
| BMDM vs. M1 | -4.281 | -6.668 to -1.894 | Yes | **** | <0.0001 |
| BMDM vs. DCs | -1.658 | -4.044 to 0.7289 | No | ns | 0.2671 |
| GM-CSF vs. M1 | -5.738 | -8.124 to -3.351 | Yes | **** | <0.0001 |
| GM-CSF vs. DCs | -3.114 | -5.501 to -0.7276 | Yes | ** | 0.0056 |
| M1 vs. DCs | 2.623 | 0.2368 to 5.010 | Yes | * | 0.0257 |
|  |  |  |  |  |  |
| H99 |  |  |  |  |  |
| BMDM vs. GM-CSF | -0.5109 | -2.897 to 1.876 | No | ns | 0.9419 |
| BMDM vs. M1 | -5.645 | -8.031 to -3.258 | Yes | **** | <0.0001 |
| BMDM vs. DCs | -2.516 | -4.903 to -0.1298 | Yes | * | 0.035 |
| GM-CSF vs. M1 | -5.134 | -7.520 to -2.747 | Yes | **** | <0.0001 |
| GM-CSF vs. DCs | -2.005 | -4.392 to 0.3811 | No | ns | 0.1294 |
| M1 vs. DCs | 3.128 | 0.7417 to 5.515 | Yes | ** | 0.0053 |
|  |  |  |  |  |  |
| KN99α |  |  |  |  |  |
| BMDM vs. GM-CSF | 0.3288 | -2.058 to 2.715 | No | ns | 0.9833 |
| BMDM vs. M1 | -4.953 | -7.339 to -2.566 | Yes | **** | <0.0001 |
| BMDM vs. DCs | -1.014 | -3.400 to 1.373 | No | ns | 0.6773 |
| GM-CSF vs. M1 | -5.282 | -7.668 to -2.895 | Yes | **** | <0.0001 |
| GM-CSF vs. DCs | -1.342 | -3.729 to 1.044 | No | ns | 0.452 |
| M1 vs. DCs | 3.939 | 1.553 to 6.326 | Yes | *** | 0.0003 |
|  |  |  |  |  |  |
| Mu-1 |  |  |  |  |  |
| BMDM vs. GM-CSF | 0.5082 | -1.878 to 2.895 | No | ns | 0.9427 |
| BMDM vs. M1 | -4.323 | -6.709 to -1.936 | Yes | **** | <0.0001 |
| BMDM vs. DCs | -3.245 | -5.631 to -0.8580 | Yes | ** | 0.0036 |
| GM-CSF vs. M1 | -4.831 | -7.218 to -2.445 | Yes | **** | <0.0001 |
| GM-CSF vs. DCs | -3.753 | -6.139 to -1.366 | Yes | *** | 0.0006 |
| M1 vs. DCs | 1.078 | -1.308 to 3.465 | No | ns | 0.633 |
|  |  |  |  |  |  |
| 24064 |  |  |  |  |  |
| BMDM vs. GM-CSF | -1.715 | -4.101 to 0.6719 | No | ns | 0.2396 |
| BMDM vs. M1 | -6.14 | -8.527 to -3.754 | Yes | **** | <0.0001 |
| BMDM vs. DCs | -2.834 | -5.221 to -0.4477 | Yes | * | 0.0137 |
| GM-CSF vs. M1 | -4.425 | -6.812 to -2.039 | Yes | **** | <0.0001 |
| GM-CSF vs. DCs | -1.12 | -3.506 to 1.267 | No | ns | 0.6044 |
| M1 vs. DCs | 3.306 | 0.9193 to 5.692 | Yes | ** | 0.0029 |

Table S5. ROS EPS Statistical analysis

| Tukey's multiple  comparisons  test | Mean  Difference. | 95.00% CI  of difference. | Below threshold? | Summary | Adjusted  P Value |
| --- | --- | --- | --- | --- | --- |
|  |  |  |  |  |  |
| *cap59Δ* |  |  |  |  |  |
| BMDM vs. GM-CSF | 0.5934 | 0.3352 to 0.8516 | Yes | **** | <0.0001 |
| BMDM vs. M1 | 0.5828 | 0.3246 to 0.8411 | Yes | **** | <0.0001 |
| BMDM vs. DCs | 0.664 | 0.4058 to 0.9222 | Yes | **** | <0.0001 |
| GM-CSF vs. M1 | -0.01056 | -0.2688 to 0.2477 | No | ns | 0.9995 |
| GM-CSF vs. DCs | 0.0706 | -0.1876 to 0.3288 | No | ns | 0.8891 |
| M1 vs. DCs | 0.08117 | -0.1771 to 0.3394 | No | ns | 0.8415 |
|  |  |  |  |  |  |
| H99 |  |  |  |  |  |
| BMDM vs. GM-CSF | 0.5251 | 0.2668 to 0.7833 | Yes | **** | <0.0001 |
| BMDM vs. M1 | 0.4173 | 0.1591 to 0.6756 | Yes | *** | 0.0004 |
| BMDM vs. DCs | 0.5931 | 0.3348 to 0.8513 | Yes | **** | <0.0001 |
| GM-CSF vs. M1 | -0.1077 | -0.3660 to 0.1505 | No | ns | 0.6921 |
| GM-CSF vs. DCs | 0.06801 | -0.1902 to 0.3263 | No | ns | 0.8995 |
| M1 vs. DCs | 0.1758 | -0.08247 to 0.4340 | No | ns | 0.2863 |
|  |  |  |  |  |  |
| Kn99α |  |  |  |  |  |
| BMDM vs. GM-CSF | 0.376 | 0.1177 to 0.6342 | Yes | ** | 0.0015 |
| BMDM vs. M1 | 0.5051 | 0.2469 to 0.7633 | Yes | **** | <0.0001 |
| BMDM vs. DCs | 0.7722 | 0.5140 to 1.030 | Yes | **** | <0.0001 |
| GM-CSF vs. M1 | 0.1291 | -0.1291 to 0.3874 | No | ns | 0.5561 |
| GM-CSF vs. DCs | 0.3963 | 0.1380 to 0.6545 | Yes | *** | 0.0008 |
| M1 vs. DCs | 0.2671 | 0.008866 to 0.5253 | Yes | * | 0.0399 |
|  |  |  |  |  |  |
| Mu-1 |  |  |  |  |  |
| BMDM vs. GM-CSF | 0.5619 | 0.3037 to 0.8202 | Yes | **** | <0.0001 |
| BMDM vs. M1 | 0.7007 | 0.4425 to 0.9590 | Yes | **** | <0.0001 |
| BMDM vs. DCs | 0.7259 | 0.4676 to 0.9841 | Yes | **** | <0.0001 |
| GM-CSF vs. M1 | 0.1388 | -0.1195 to 0.3970 | No | ns | 0.4949 |
| GM-CSF vs. DCs | 0.1639 | -0.09430 to 0.4222 | No | ns | 0.3469 |
| M1 vs. DCs | 0.02516 | -0.2331 to 0.2834 | No | ns | 0.994 |
|  |  |  |  |  |  |
| 24064 |  |  |  |  |  |
| BMDM vs. GM-CSF | 0.4485 | 0.1903 to 0.7067 | Yes | *** | 0.0001 |
| BMDM vs. M1 | 0.6031 | 0.3449 to 0.8614 | Yes | **** | <0.0001 |
| BMDM vs. DCs | 0.5872 | 0.3289 to 0.8454 | Yes | **** | <0.0001 |
| GM-CSF vs. M1 | 0.1546 | -0.1036 to 0.4129 | No | ns | 0.3991 |
| GM-CSF vs. DCs | 0.1387 | -0.1196 to 0.3969 | No | ns | 0.4956 |
| M1 vs. DCs | -0.01596 | -0.2742 to 0.2423 | No | ns | 0.9985 |
|  |  |  |  |  |  |
| C |  |  |  |  |  |
| BMDM vs. GM-CSF | 0.06175 | -0.1965 to 0.3200 | No | ns | 0.9223 |
| BMDM vs. M1 | 0.2716 | -0.007311 to 0.5505 | No | ns | 0.0591 |
| BMDM vs. DCs | 0.3028 | 0.04456 to 0.5610 | Yes | * | 0.015 |
| GM-CSF vs. M1 | 0.2099 | -0.06906 to 0.4888 | No | ns | 0.2054 |
| GM-CSF vs. DCs | 0.2411 | -0.01719 to 0.4993 | No | ns | 0.076 |
| M1 vs. DCs | 0.03118 | -0.2477 to 0.3101 | No | ns | 0.9911 |

Table S6. ROS EPS+β-Glucan Statistical analysis

| Tukey's multiple  comparisons  test | Mean  Difference. | 95.00% CI  of difference. | Below threshold? | Summary | Adjusted  P Value |
| --- | --- | --- | --- | --- | --- |
|  |  |  |  |  |  |
| *cap59Δ* |  |  |  |  |  |
| BMDM vs. GM-CSF | 0.6334 | 0.3766 to 0.8903 | Yes | **** | <0.0001 |
| BMDM vs. M1 | 0.5462 | 0.2894 to 0.8030 | Yes | **** | <0.0001 |
| BMDM vs. DCs | 0.1646 | -0.09221 to 0.4215 | No | ns | 0.3386 |
| GM-CSF vs. M1 | -0.08725 | -0.3441 to 0.1696 | No | ns | 0.8082 |
| GM-CSF vs. DCs | -0.4688 | -0.7257 to -0.2120 | Yes | **** | <0.0001 |
| M1 vs. DCs | -0.3816 | -0.6384 to -0.1247 | Yes | ** | 0.0012 |
|  |  |  |  |  |  |
| H99 |  |  |  |  |  |
| BMDM vs. GM-CSF | 0.3633 | 0.1065 to 0.6201 | Yes | ** | 0.0022 |
| BMDM vs. M1 | 0.4655 | 0.2087 to 0.7223 | Yes | **** | <0.0001 |
| BMDM vs. DCs | 0.4303 | 0.1735 to 0.6872 | Yes | *** | 0.0002 |
| GM-CSF vs. M1 | 0.1022 | -0.1546 to 0.3590 | No | ns | 0.7227 |
| GM-CSF vs. DCs | 0.06702 | -0.1898 to 0.3239 | No | ns | 0.902 |
| M1 vs. DCs | -0.03518 | -0.2920 to 0.2216 | No | ns | 0.9839 |
|  |  |  |  |  |  |
| Kn99α |  |  |  |  |  |
| BMDM vs. GM-CSF | 0.4452 | 0.1884 to 0.7020 | Yes | *** | 0.0001 |
| BMDM vs. M1 | 0.6977 | 0.4409 to 0.9545 | Yes | **** | <0.0001 |
| BMDM vs. DCs | 0.9409 | 0.6841 to 1.198 | Yes | **** | <0.0001 |
| GM-CSF vs. M1 | 0.2525 | -0.004319 to 0.5093 | No | ns | 0.0557 |
| GM-CSF vs. DCs | 0.4957 | 0.2389 to 0.7526 | Yes | **** | <0.0001 |
| M1 vs. DCs | 0.2432 | -0.01361 to 0.5001 | No | ns | 0.0699 |
|  |  |  |  |  |  |
| Mu-1 |  |  |  |  |  |
| BMDM vs. GM-CSF | 0.2736 | 0.01673 to 0.5304 | Yes | * | 0.0324 |
| BMDM vs. M1 | 0.5845 | 0.3277 to 0.8414 | Yes | **** | <0.0001 |
| BMDM vs. DCs | 0.7698 | 0.5130 to 1.027 | Yes | **** | <0.0001 |
| GM-CSF vs. M1 | 0.311 | 0.05412 to 0.5678 | Yes | * | 0.0113 |
| GM-CSF vs. DCs | 0.4963 | 0.2394 to 0.7531 | Yes | **** | <0.0001 |
| M1 vs. DCs | 0.1853 | -0.07152 to 0.4421 | No | ns | 0.2382 |
|  |  |  |  |  |  |
| 24064 |  |  |  |  |  |
| BMDM vs. GM-CSF | 0.1922 | -0.06461 to 0.4491 | No | ns | 0.2096 |
| BMDM vs. M1 | 0.6979 | 0.4410 to 0.9547 | Yes | **** | <0.0001 |
| BMDM vs. DCs | 0.6263 | 0.3694 to 0.8831 | Yes | **** | <0.0001 |
| GM-CSF vs. M1 | 0.5056 | 0.2488 to 0.7625 | Yes | **** | <0.0001 |
| GM-CSF vs. DCs | 0.434 | 0.1772 to 0.6909 | Yes | *** | 0.0002 |
| M1 vs. DCs | -0.07161 | -0.3284 to 0.1852 | No | ns | 0.8834 |
|  |  |  |  |  |  |
| C |  |  |  |  |  |
| BMDM vs. GM-CSF | -0.3164 | -0.5732 to -0.05955 | Yes | ** | 0.0096 |
| BMDM vs. M1 | 0.113 | -0.1438 to 0.3699 | No | ns | 0.6553 |
| BMDM vs. DCs | 0.1583 | -0.09853 to 0.4151 | No | ns | 0.3734 |
| GM-CSF vs. M1 | 0.4294 | 0.1726 to 0.6863 | Yes | *** | 0.0002 |
| GM-CSF vs. DCs | 0.4747 | 0.2179 to 0.7315 | Yes | **** | <0.0001 |
| M1 vs. DCs | 0.04526 | -0.2116 to 0.3021 | No | ns | 0.9667 |


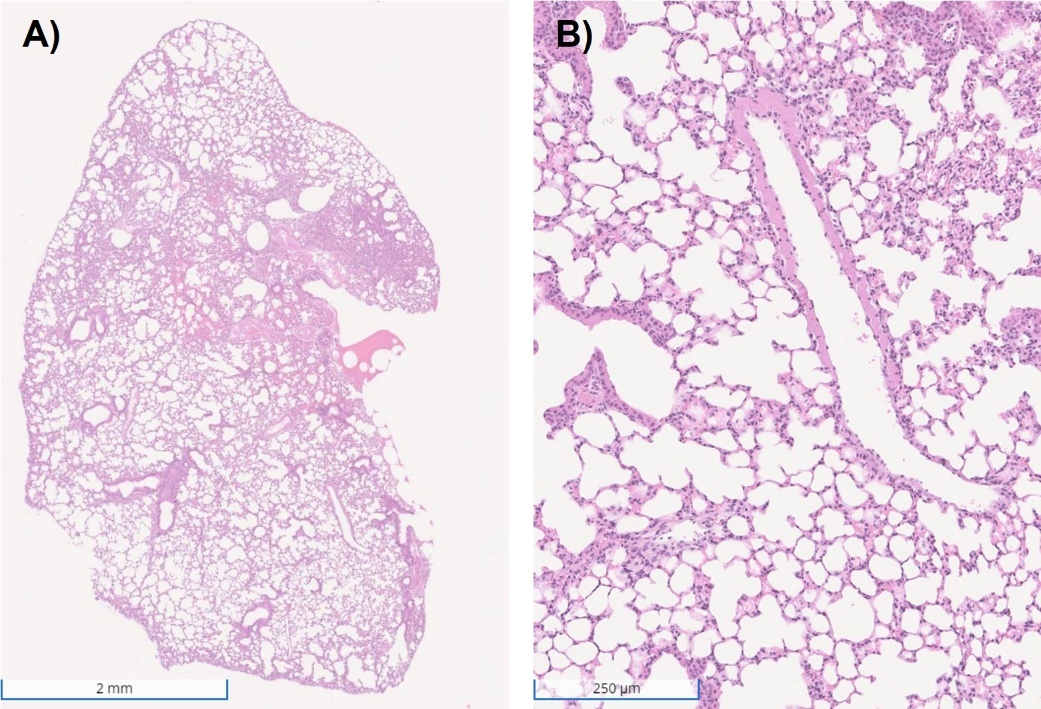


Fig. S8. Histological findings SHAM (non-infected). After euthanasia randomly selected lungs had a small tissue sample piece aseptically excised and preserved in formalin until analysis. A) Sham 1× magnification. B) Sham 6× magnification. HE stain.
